# Pleiotrophin regulates presynaptic assembly and function through heparan sulfate-dependent binding to neurexin1

**DOI:** 10.1016/j.celrep.2026.117827

**Published:** 2026-08-12

**Authors:** Qin Xu, Andrew W. Schilling, Alexander W. Sorum, Peipei Zhang, Destini C. Weller, Donovan Whitfield, Gokul Velayoudame, Allison J. Wu, Qian Sun, Linda C. Hsieh-Wilson, Peng Zhang

**Affiliations:** 1 Department of Neurosciences, School of Medicine, Case Western Reserve University, Cleveland, OH 44106, USA; 2 Division of Chemistry and Chemical Engineering, California Institute of Technology, 1200 E. California Blvd, Pasadena, CA 91125, USA; 3 Lead contact

## Abstract

Synapses are the fundamental units of neural circuits, and their dysfunction contributes to numerous neuropsychiatric disorders. Although synaptic adhesion proteins have been well studied, how extracellular cues and matrix glycans specify synaptic properties remains much less well understood. Here, we identify pleiotrophin (Ptn) as a regulator of presynaptic development. Affinity purification-based proteomics shows that Ptn associates with heparan sulfate (HS)-modified neurexin1 (HS-Nrxn1) in the brain through an HS-glycan-dependent mechanism. Glycan microarray analyses further reveal that Ptn selectively recognizes defined HS sulfation motifs. Functionally, Ptn requires both HS glycans and Nrxns to induce presynaptic assembly in cultured neurons. *In vivo*, Ptn deletion disrupts presynaptic protein clustering, reduces neurotransmitter release probability at CA3-CA1 synapses, and impairs contextual fear discrimination. Together, these findings establish Ptn as an extracellular organizer of presynaptic development and support a model in which Nrxn1’s HS glycan provides a platform for extracellular ligand recruitment.

## In brief

Xu et al. identify pleiotrophin as a heparan sulfate-dependent binding partner of neurexin1 and show that pleiotrophin regulates presynaptic assembly and function, uncovering an extracellular glycan-dependent pathway that links soluble matrix factors to synapse organization.

## Graphical Abstract

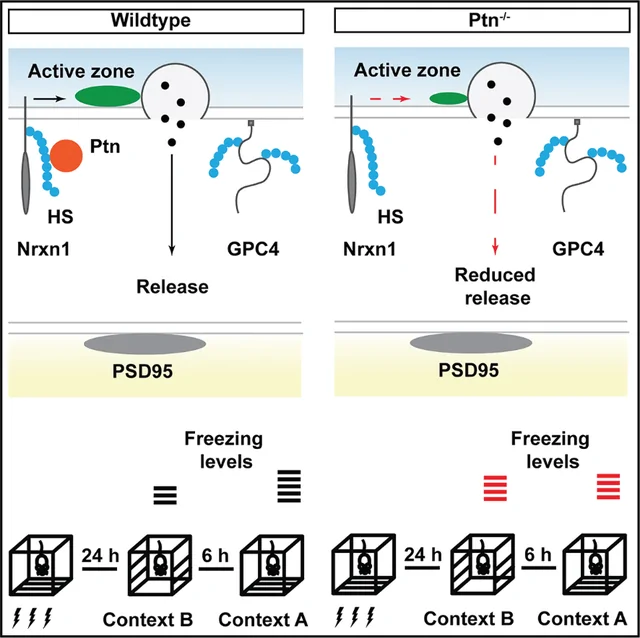

## INTRODUCTION

Synapses are the fundamental units of neural circuits, and their dysfunction is a hallmark of numerous neuropsychiatric and neurodegenerative disorders. Although extensive work has established the roles of *trans*-synaptic adhesion molecules in synapse development, much less is understood about how extracellular cues and complex glycans shape the assembly and specialization of synaptic properties.^1^

Heparan sulfate proteoglycans (HSPGs) are proteins decorated with heparan sulfate (HS), one major type of extracellular matrix glycans.^2^ In addition to their classic roles in early brain development, HSPGs are emerging as critical regulators of synapse development.^3,4^ For example, the canonical synaptic organizing proteins neurexins (Nrxns) have been recently identified as HSPGs, and selective deletion of HS from Nrxn1 results in deficits in both presynaptic and postsynaptic structures and function.^5^ Another HSPG, glypican-4 (GPC4), is essential for maintaining normal synaptic AMPA receptor levels at hippocampal excitatory synapses.^6^ These findings highlight the critical yet diverse roles of individual HSPGs in regulating the organization and function of presynaptic or postsynaptic terminals.

Several synaptic organizers, such as LRRTM4 and GPR158, interact with HS glycans.^7–9^ However, their genetic disruption primarily impairs postsynaptic function,^7–9^ raising the question of which HS-binding factors mediate presynaptic development. Furthermore, HS glycans undergo position-specific sulfation and thereby generate thousands of potential sulfation motifs with distinct binding properties.^10^ It is also unknown whether synaptic organizers recognize particular HS sulfation motifs.

Pleiotrophin (Ptn) has been identified as a secreted cytokine and growth factor because it is acutely upregulated following brain injury or inflammation-associated neurological disorders, including drug addiction, Parkinson disease, multiple sclerosis, and Alzheimer disease.^11–16^ More recently, dysregulated Ptn expression has been implicated in Down syndrome, depression, and mania.^17–20^ Despite its broad relevance to disease, the physiological functions of Ptn during brain development remain poorly understood. As a secreted protein, Ptn must act through receptors. Most of its known functions have been attributed to its best-characterized receptor, protein tyrosine phosphatase receptor ζ1 (Ptprz1), which mediates Ptn-dependent signaling in the proliferation of neurons, astrocytes, and oligodendrocyte precursor cells.^17,18,20–22^ However, a systematic identification of endogenous Ptn-binding partners in the brain has been lacking. Because Ptn can bind to HS glycans *in vitro*,^23^ and because we previously showed that Ptn interacts with recombinant Nrxn1β via HS in cultured neurons,^5^ we hypothesized that Ptn may act through HS-modified receptors, including Nrxn1, to execute its diverse functions in the brain.

Here, we systematically address these open questions using proteomic, biochemical, genetic, and functional approaches. We find that Ptn forms endogenous complexes with HS-modified Nrxn1 *in vivo*, showing strong selectivity for Nrxn1 over other abundant brain HSPGs. HS glycan microarray analysis further reveals that Ptn recognizes defined sulfation motifs, suggesting that Ptn interactions with HSPGs such as Nrxn1 are governed by precise sulfation patterning, rather than the overall sulfate density. Functionally, Ptn requires both HS glycans and Nrxns to induce presynaptic assembly in cultured neurons. *In vivo*, loss of Ptn disrupts presynaptic active zone organization and neurotransmitter release probability at hippocampal CA3-CA1 synapses and leads to deficits in contextual fear discrimination. Together, these findings identify Ptn as a distinct HS-dependent synaptic organizer that engages HS-modified Nrxn1 to regulate presynaptic development and function. More broadly, our studies uncover a mechanism by which extracellular glycan- mediated cues instruct specific aspects of synapse development and function.

## RESULTS

### Proteomic profiling identifies synapse-related pathways and Nrxns as major Ptn-binding partners

**Figure 1.**
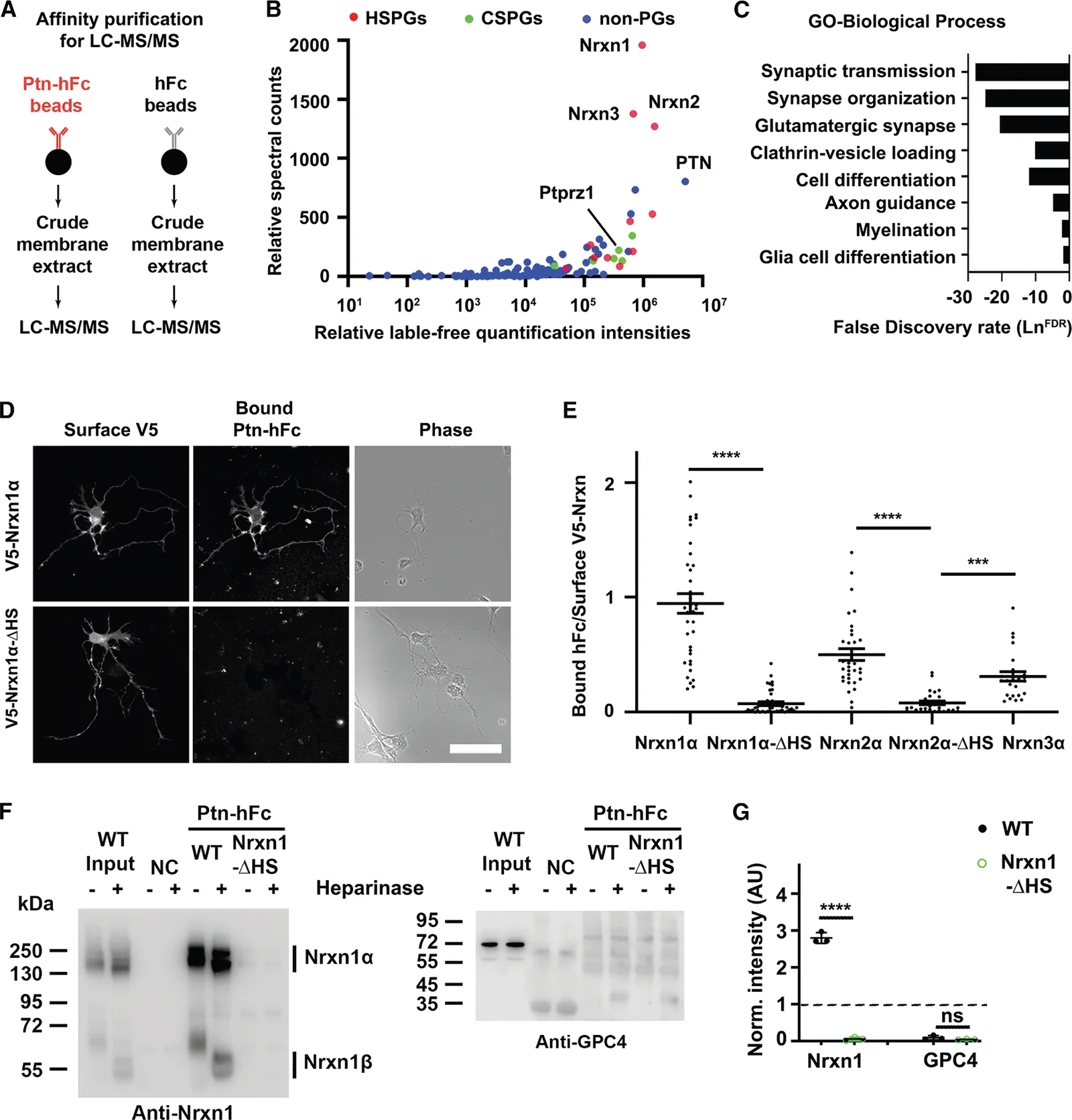
Proteomic identification of Ptn-interacting proteins and HS-dependent binding to Nrxns (A) Schematic of the affinity purification LC-MS/MS workflow, using Ptn-hFc or hFc control beads to isolate interacting proteins from mouse brain crude membrane extracts. (B) Scatterplot showing relative spectral counts versus label-free quantification (LFQ) intensities of identified proteins after Fc subtraction. Proteins are categorized as HSPGs, CSPGs, and non-proteoglycans. (C) Gene Ontology (GO) analysis of Ptn-interacting proteins, highlighting enriched biological processes. FDR, false discovery rate. (D) Representative images of Ptn-hFc binding to V5-Nrxn1α-WT but not to HS-deficient (ΔHS) mutant, expressed in primary rat hippocampal neurons. Note that Ptn-hFc failed to bind to non-transfected neurons shown in phase images. Scale bars, 50 μm. (E) Quantification of Ptn-hFc binding to V5-tagged Nrxn1α, Nrxn1α-ΔHS, Nrxn2α, Nrxn2α-ΔHS, and Nrxn3α. One-way ANOVA with Bonferroni’s tests (****p < 0.0001; ***p < 0.001; Nrxn1α binding was significantly greater than Nrxn2α or Nrxn3α binding (*p* < 0.0001), whereas binding to Nrxn2α was not significantly different from binding to Nrxn3α (*p* = 0.16). n = 25–43 cells in 3 independent experiments. (F and G) Heparinase treatment enables quantitative comparison of Ptn-associated HSPGs. (F) Immunoblot of Nrxn1 and GPC4 pulled down by Ptn-hFc from WT and Nrxn1ΔHS mouse brain lysates. Heparinase treatment was applied after pull-down and washing, once protein complexes had formed, to collapse heterogeneous HS-modified smears into discrete core protein bands for accurate quantification. hFc beads were used as negative control (NC). (G) Enrichment of Nrxn1 and GPC4 was quantified as fold change relative to input (dashed line). Nrxn1 shows strong enrichment in WT samples that is abolished in Nrxn1ΔHS mice, whereas GPC4 does not show enrichment. One-way ANOVA with Bonferroni’s test, comparing WT input with WT pull-down (Nrxn1 *p* < 0.0001; GPC4 *p* < 0.0001) and comparing pull-down between WT and ΔHS (****p < 0.0001; ns, not significant); n = 3 independent experiments. Error bars, SEM. See also Figure S1 and Tables S1 and S2.

To identify Ptn-binding partners in the brain, we performed affinity purification using Ptn-hFc and hFc as a control with solubilized crude membrane fractions from mouse cortical and hippocampal tissues. Bound proteins were analyzed by liquid chromatography-tandem mass spectrometry (LC-MS/MS) (Figure 1A). In a plot by relative spectral counts and peptide intensities, Nrxns were among the most strongly enriched proteins in the Ptn pull down, while other HSPGs and chondroitin sulfate proteoglycans (CSPGs) were also detected, including Ptprz1, the best-known Ptn receptor (Figure 1B; Tables S1 and S2). This result was notable, as previous LRRTM4-hFc proteomic screens showed a strong preference for glypicans (GPCs) over Nrxns,^8,9^ suggesting that Ptn may differ in its selective association toward Nrxns in the brain. Gene Ontology (GO) analysis further revealed that the enriched proteins were associated with pathways related to synaptic transmission and organization (Figure 1C), supporting the potential role of Ptn in synapse development.

To determine whether Ptn interacts differentially with Nrxn isoforms, we examined the binding of Ptn-hFc to recombinant V5-tagged Nrxn1α, Nrxn2α, and Nrxn3α expressed in primary neurons. Ptn-hFc bound to all three Nrxn isoforms, with a higher binding observed for Nrxn1α (Figures 1D and 1E; Figures S1A and S1B). This interaction depended on HS modification, as binding to HS-deficient Nrxn mutants (ΔHS) was substantially reduced. In addition, Ptn-hFc did not bind to non-transfected neurons under the same conditions, confirming the specificity of this interaction. Together, these results indicate that Ptn can engage multiple Nrxn isoforms but shows stronger binding to HS-modified Nrxn1 in cultured neurons.

Because HS biosynthesis and modification are cell type dependent,^2,24^ we next asked whether this preferential interaction is recapitulated in the brain. To test this, we performed Ptn-hFc pull-down assays using mouse brain lysates to assess its association with different HSPGs. Brain lysates from Nrxn1-ΔHS mice were used to determine whether the Ptn-Nrxn1 interaction depends on HS modification of Nrxn1. To enable accurate comparisons across targets, pull-down signals were normalized to input levels to account for differences in protein abundance, and heparinase treatment was applied after pull down to collapse HS-modified smears into discrete core protein bands for accurate quantification. Accordingly, HS dependence was assessed genetically using the Nrxn1-ΔHS mouse line rather than through enzymatic HS removal.

Under these conditions, Ptn-hFc efficiently pulled down endogenous Nrxn1 from WT brain lysates while showing minimal enrichment of GPC4, and this interaction was strongly reduced in Nrxn1-ΔHS mice (Figures 1F and 1G). These findings were further supported by immunoblotting with pan-Nrxn antibodies and were not attributable to differences in expression of Nrxn1 between WT and Nrxn1-ΔHS mice (Figures S1C–S1F). Together, these findings suggest that Ptn associates more strongly with Nrxn1 than with GPC4 in the brain.

### HS modification of Nrxn1 is required for Ptn-Nrxn1 complex formation *in vivo*

**Figure 2.**
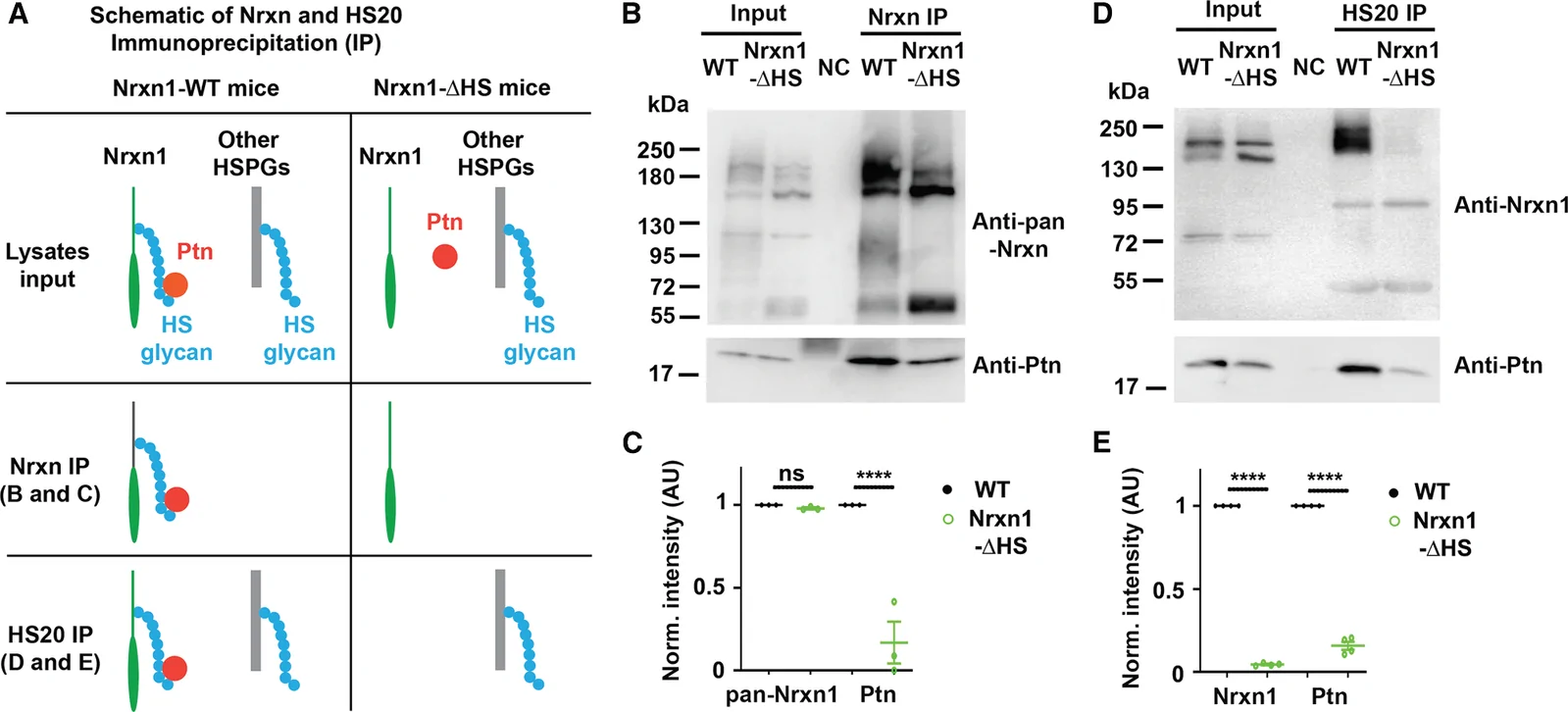
HS-dependent interaction between endogenous Ptn and Nrxn1 in the brain (A) Schematic of the immunoprecipitation strategies used to examine interactions between Ptn and HS-modified proteins in WT and Nrxn1ΔHS mouse brains. (B) Immunoprecipitation of Nrxns from brain lysates, followed by immunoblotting for Nrxn1 and Ptn. Beads only were used as negative control (NC). Endogenous Ptn co-immunoprecipitation with Nrxn1 was reduced in Nrxn1ΔHS mice. (C) Relative intensities of Nrxn1 and Ptn that were immunoprecipitated between WT and Nrxn1ΔHS mouse brains. One-way ANOVA with Bonferroni’s test (****p < 0.0001; ns, not significant); n = 3 independent experiments. (D) HS20 immunoprecipitation of HSPGs, followed by immunoblotting for Nrxn1 and Ptn. Human IgG was used as negative control (NC). Ptn association with HSPGs was reduced in Nrxn1ΔHS mice. (E) Relative intensities of Nrxn1 and Ptn that were immunoprecipitated between WT and Nrxn1ΔHS mouse brains. One-way ANOVA with Bonferroni’s test (****p < 0.0001); n = 4 independent experiments. Error bars, SEM. See also Figure S2.

We next examined whether Ptn forms an endogenous complex with HS-modified Nrxn1 in the mouse brain, using two complementary immunoprecipitation approaches (Figure 2A). In the first approach, Nrxns were immunoprecipitated to assess endogenous Ptn-Nrxn complex formation. Endogenous Ptn was readily detected in Nrxn immunoprecipitates from WT samples, whereas its association was nearly abolished in Nrxn1-ΔHS mice, despite comparable recovery of Nrxn1 proteins (Figures 2B and 2C). These results are consistent with a requirement for HS modification of Nrxn1 in mediating this interaction.

In the second approach, HS-modified proteoglycans were isolated using the HS-specific antibody HS20^25^ to evaluate the contribution of Nrxn1 HS to Ptn association within the broader pool of brain HSPGs. As expected, Nrxn1 was detected in HS20 precipitates from WT but not in those from Nrxn1-ΔHS mice, confirming the enrichment of HS-modified Nrxn1 by this approach (Figures 2D and 2E). Notably, Ptn signal in HS20 precipitates was strongly reduced in Nrxn1-ΔHS mice compared with WT, despite comparable recovery of total HSPGs (Figure S2). Thus, although Nrxn1 constitutes only a minor fraction of total brain HSPGs, it contributes disproportionately to Ptn association within the detectable HS-modified proteoglycan pool.

Together, these results demonstrated that HS modification of Nrxn1 is required for Ptn-Nrxn1 complex formation and identifies Nrxn1 as a prominent binding partner for Ptn among brain HSPGs under these experimental conditions. The findings suggest that Ptn binding is not determined solely by the presence of HS, but instead may depend on specific HS sulfation motifs present on Nrxn1.

### Ptn recognizes specific HS sulfation motifs

**Figure 3.**
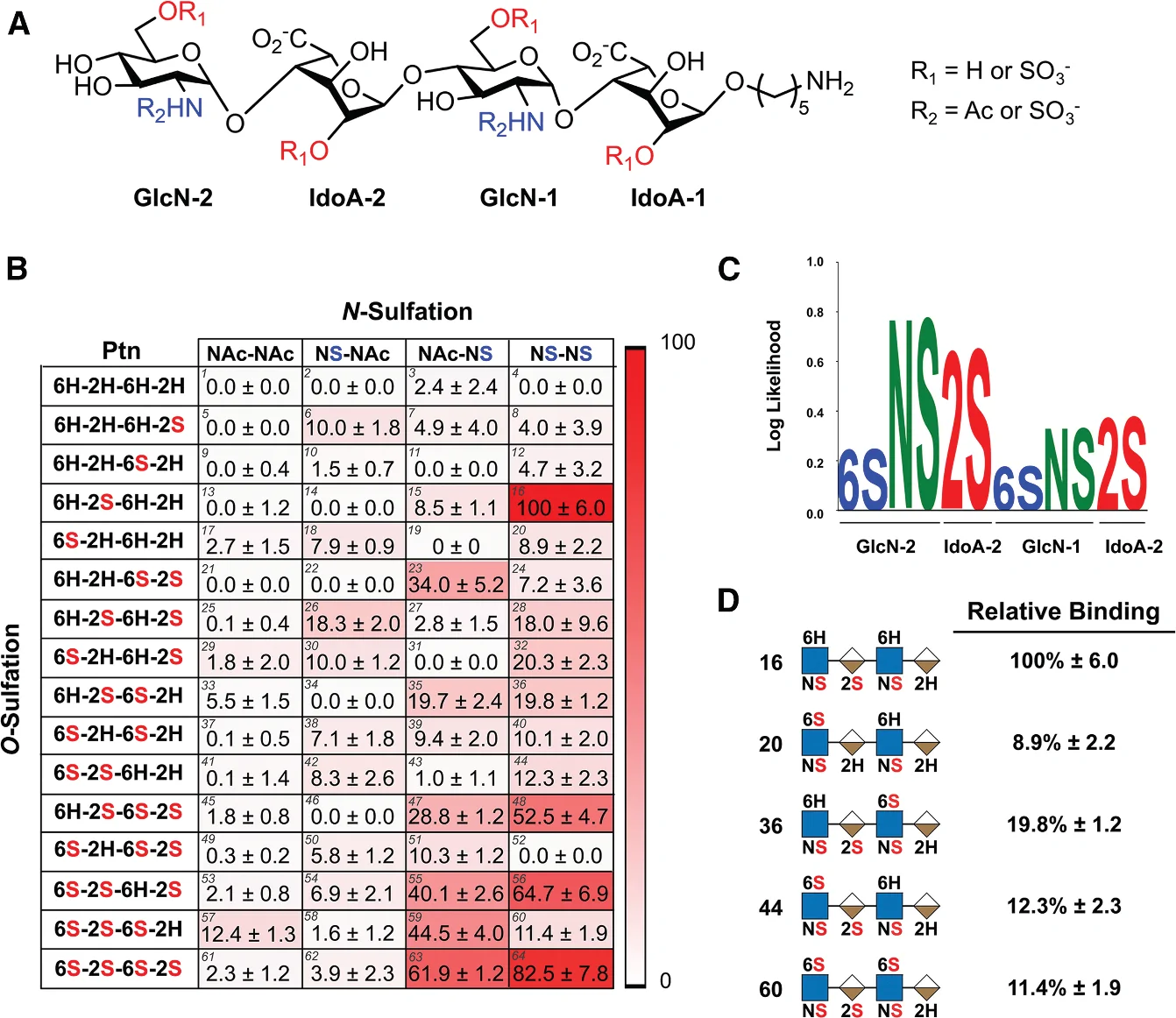
Ptn preferentially recognizes specific sulfated HS structures (A) Schematic of a 64-member heparan sulfate (HS) tetrasaccharide library containing all possible sulfation patterns at the most commonly modified positions (2-*O*, 6-*O*, and N sites). (B) Heatmaps showing relative binding of Ptn-hFc to each member of the HS library. Fluorescence intensities were baseline corrected and normalized with respect to the greatest binding signal. Data are represented as the mean ± SD. Experiments were performed in duplicate. (C) Sulfation logos of Ptn-hFc were computationally generated from the microarray data. Logos depict enrichment of the modification on a logarithmic scale at each position in the bound sequences. 6S, 6-*O*-sulfation; NS, N-sulfation; 2S, 2-*O*-sulfation. (D) Ptn-hFc exhibits distinct affinities toward HS tetrasaccharides containing similar levels but different patterns of sulfation. See also Figure S3.

To investigate whether Ptn recognizes particular HS sulfation patterns, we employed glycan microarrays containing a comprehensive 64-compound HS tetrasaccharide library spanning all combinations of 2-*O*, 6-*O*, and N-sulfation within the D-glucosamine (GlcN)-L-iduronic acid (IdoA)-GlcN-IdoA backbone (Figure 3A).^26^ Binding of Ptn-hFc to the HS microarray was detected using an Alexa Fluor 647-conjugated anti-hFc antibody and visualized as a heatmap (Figure 3B), bar graph (Figure S3), and sulfation frequency logos (Figure 3C).^26^ Ptn exhibited striking selectivity for specific HS sequences, preferentially binding structures enriched in N-sulfation and 2-*O*-sulfation while generally disfavoring 6-*O*- sulfation (Figures 3B and 3C). Importantly, Ptn binding did not correlate simply with the overall sulfate density. For example, the tri-sulfated compound **16** (GlcNS-IdoA2S-GlcNS-IdoA) emerged as the highest binder on the microarray, despite not being the most highly sulfated structure (Figure 3D). Remarkably, repositioning the sulfate group from the 2-*O*-position of IdoA-2 to the 6-*O*-position of GlcN-2 (compound **20**, GlcNS,6S-IdoA-GlcNS-IdoA) reduced Ptn binding from 100% to 8.9%. Similarly, addition of 6-*O*-sulfate groups to compound **16** at GlcN-1 (**36**, GlcNS-IdoA2S-GlcNS,6S-IdoA), GlcN-2 (**44**, GlcNS,6S-IdoA2S-GlcNS-IdoA), or both positions (**60**, GlcNS,6S-IdoA2S- GlcNS,6S-IdoA) reduced binding to 20%, 12%, and 11%, respectively, despite increasing overall negative charge. Together, these results demonstrated that Ptn-HS interactions are governed not simply by sulfate density but by precise sulfation patterning and positioning within the HS sequence. Thus, Ptn recognizes specific HS sulfation motifs in the absence of the Nrxn1 core protein.

### Ptn requires both HS glycan and Nrxns to induce presynaptic assembly

**Figure 4.**
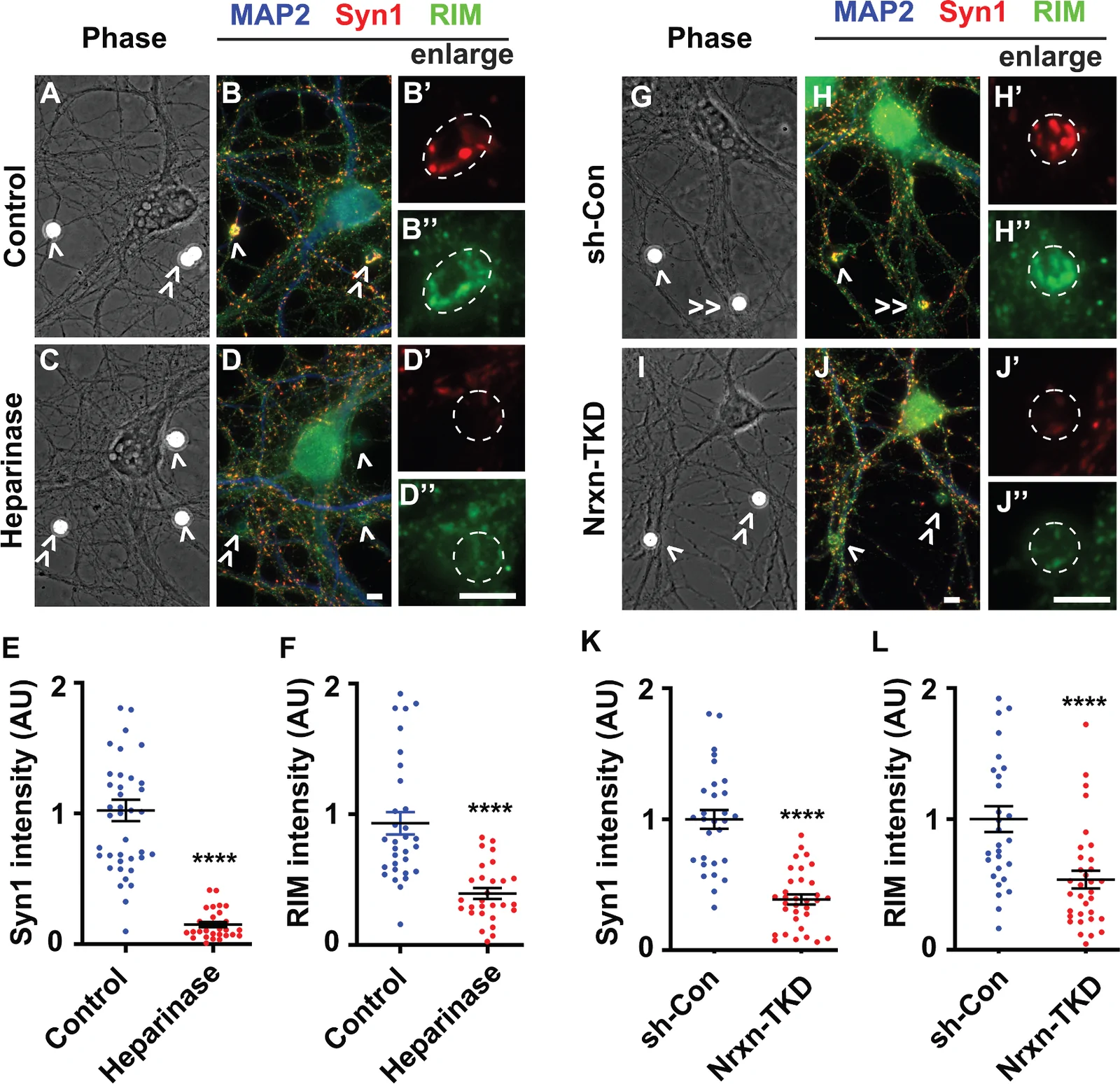
Ptn requires HS and Nrxns to induce presynaptic assembly (A–D″) Representative images of hippocampal neurons incubated with Ptn-hFc beads, showing recruitment of Syn1 and RIM under control conditions (A and B′′) or following heparinase treatment (C and D″). (E and F) Quantification of Syn1 and RIM intensity at Ptn-hFc bead-axon contact sites. Heparinase treatment significantly reduces recruitment. Mann-Whitney or Welch’s *t* test; ****p < 0.0001; n = 30–40 from 3 experiments. (G–J″) Representative images showing reduced Syn1 and RIM recruitment following Nrxn knockdown (Nrxn-TKD). (K and L) Quantification of Syn1 and RIM intensity under control and Nrxn knockdown conditions. Mann-Whitney or Welch’s test; ****p < 0.0001; n = 29–44 from 3 experiments. Error bars, SEM. Scale bars, 20 μm. See also Figure S4.

To determine the functional consequences of Ptn-HS-Nrxn interactions, we examined whether Ptn promotes presynaptic assembly in cultured neurons, using a bead-neuron co-culture assay. Ptn-hFc beads efficiently recruited presynaptic markers, including synapsin-1 (Syn1) and the active zone protein RIM, to the bead-neuron contact sites (Figures 4A and 4B″).

To test whether this activity depends on HS, neurons were treated with heparinase to remove cell-surface HS. Effective removal of HS by heparinase was confirmed by increased 3G10 reactivity (Figure S4I). Heparinase treatment abolished Ptn-induced clustering of Syn1 and RIM (Figures 4C and 4D″), and quantification confirmed a significant reduction in presynaptic assembly (Figures 4E and 4F). These results indicated that cell-surface HS is required for Ptn-induced presynaptic differentiation.

We next asked whether Nrxns mediate this effect. Because Nrxns function redundantly in cultured neurons^5,27^ and Ptn binds to multiple Nrxn isoforms (Figures 1D and 1E; Figure S1A), we hypothesized that Ptn engages Nrxns for presynaptic assembly. To test this, we knocked down all Nrxn family members, using pre-validated small hairpin RNAs (shRNAs)^5,27,28^ delivered by adeno-associated viruses (AAVs). Nrxn knockdown markedly reduced Nrxn signals and presynaptic markers, including vGlut1, Syn1, and RIM, surrounding Ptn-hFc beads (Figures 4G–4L; Figures S4A–S4H).

Together, these results demonstrated that Ptn promotes presynaptic assembly and that both HS and Nrxns are required in this assay, consistent with a model in which Ptn acts through HS-dependent interactions with Nrxns to promote presynaptic assembly.

### Ptn is enriched at synapses but does not regulate spine density in CA1 neurons

To assess the *in vivo* role of Ptn, we first validated Ptn deletion by immunoblotting in a previously generated Ptn^−/−^ mouse line (Figure S5A). We next examined Ptn expression during postnatal development and found that Ptn levels peak at early postnatal stages and decline in adulthood (Figure S5B). Notably, this temporal profile coincides with this process of synapse development in the mouse hippocampus, supporting a role for Ptn in synapse development. To determine whether Ptn is localized to synaptic compartments, we analyzed its enrichment in the synaptic plasma membrane (SPM) fractions. Ptn was significantly enriched in SPM fractions relative to homogenate in WT mice, whereas this enrichment was reduced in Nrxn1-ΔHS mice (Figure S5C), consistent with a contribution of Nrxn1’s HS to Ptn localization at synapses.

Ptn deletion did not affect the overall body weight in either male mice or female mice (Figure S5D). We next examined the dendritic spine density of hippocampal CA1 pyramidal neurons, where Ptn mRNA is highly expressed (Allen Institute ISH and Cembrowski et al.^29^). Spine density was comparable between the WT and Ptn^−/−^ mice (Figure S5E), suggesting that Ptn is not required for spine formation in this region.

### Ptn is required for presynaptic assembly *in vivo*

**Figure 5.**
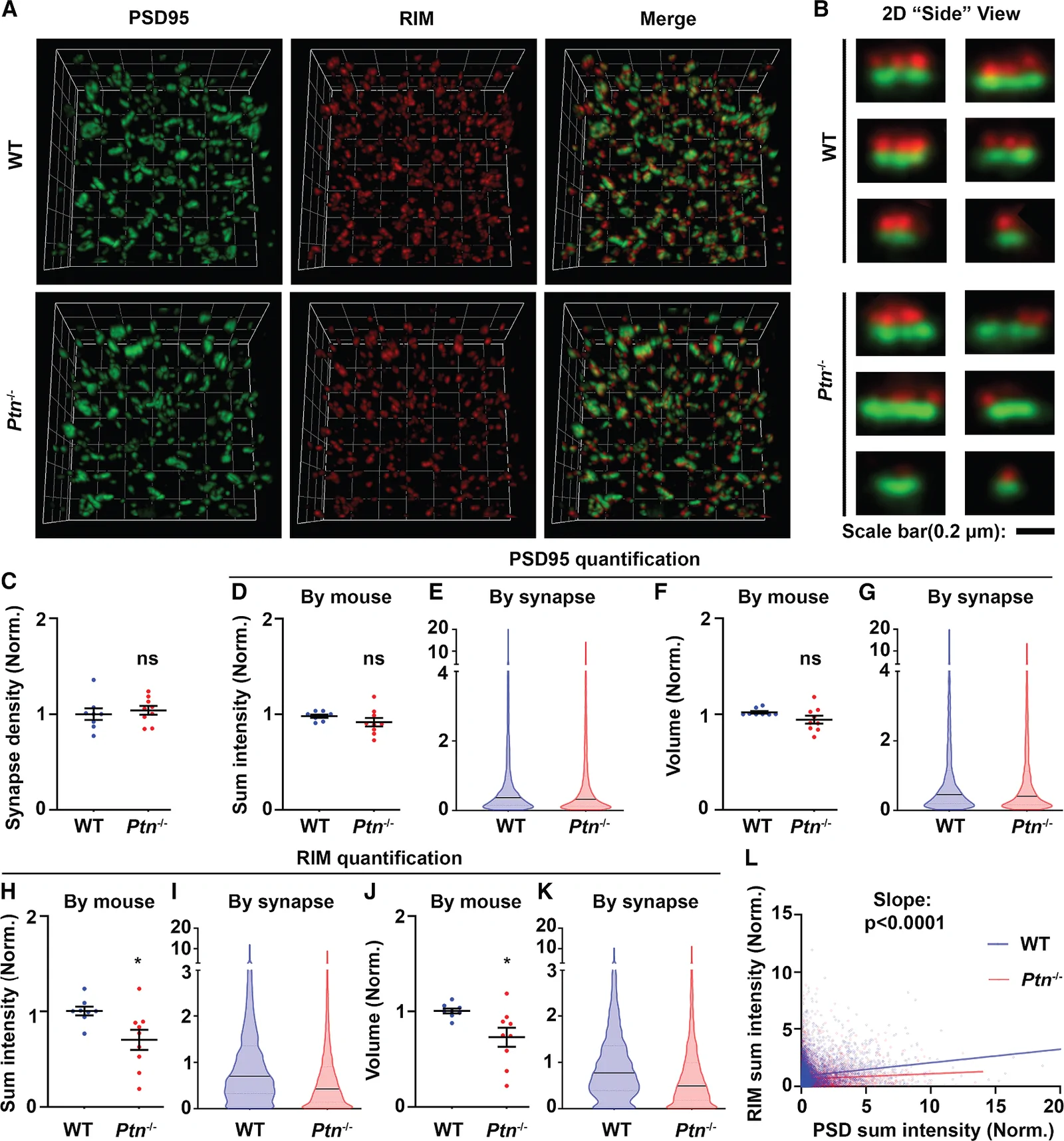
Ptn^−/−^ mice show impaired RIM clustering *in vivo* (A) Representative 3D expansion microscopy images of PSD95 and RIM in CA1 stratum radiatum from WT and Ptn^−/−^ mice. 3D volume: 5 × 5 × 2.5 μm (*x* × *y* × *z*). (B) Example 2D images of individual synapses. Scale bars, 0.2 μm. (C–E) Quantification of PSD95 synapse density, intensity, and volume shows no significant differences between genotypes. (F–K) Quantification of RIM intensity and volume reveals reduced presynaptic organization in Ptn^−/−^ mice. (L) Relationship between PSD95 and RIM intensity at individual synapses. Slopes of linear regression lines differ significantly between WT and Ptn^−/−^ mice (*p* < 0.0001). *p < 0.05; ns, not significant, by unpaired *t* test or Mann-Whitney test; n = 5,962 synapses from 8 WT mice and 6,863 synapses from 9 Ptn^−/−^ mice. Individual synapse data are shown to illustrate distributions and were not used as independent statistical units. Statistical analyses were performed using mice as biological replicates. Error bars, SEM.

Although the spine density was unchanged, we further determined alterations in the presynaptic protein machinery, such as RIM, which can also impair synaptic function.^30^ Because Ptn can recruit RIM *in vitro* (Figure 4), we analyzed RIM expression in the WT and Ptn^−/−^ mice. Conventional light microscopy is difficult to reliably resolve RIM proteins at single synapses in intact brain tissues. Therefore, we used a modified magnified analysis of the proteome (MAP) method^31^ to quantify RIM proteins at individual synapses in CA1 regions of mouse hippocampus (Figures 5A and 5B). Synapses were defined as PSD95^+^ puncta apposed to RIM clusters. Because individual synapses are highly heterogeneous,^32^ we quantified signals at the level of individual synapses and averaged them per animal. Statistical analyses were performed using individual mice as biological replicates; synapse-level data are shown to illustrate the distribution of synaptic proteins.

Ptn deletion did not alter CA3-CA1 synapse density (Figure 5C), consistent with the unchanged spine density. Moreover, postsynaptic density (PSD) sum intensity and volume per synapse were not altered across mice (Figures 5D–5G). In contrast, both RIM intensity and volume per synapse were significantly reduced in Ptn^−/−^ mice compared with WT (Figures 5H–5K). Consistent with previous super-resolution imaging in cultured neurons,^32^ we observed heterogeneity in RIM and PSD95 signals across synapses. Notably, Ptn^−/−^ mice exhibited a spectrum of variable severity in RIM (Figure 5B). To account for this variability, we plotted RIM intensity against PSD95 intensity across individual synapses (Figure 5L). We found that the rate at which RIM intensity increases with PSD intensity was significantly lower in Ptn^−/−^ mice than in WT mice, supporting a role for Ptn in presynaptic assembly.

### Ptn deletion impairs excitatory synaptic transmissions

**Figure 6.**
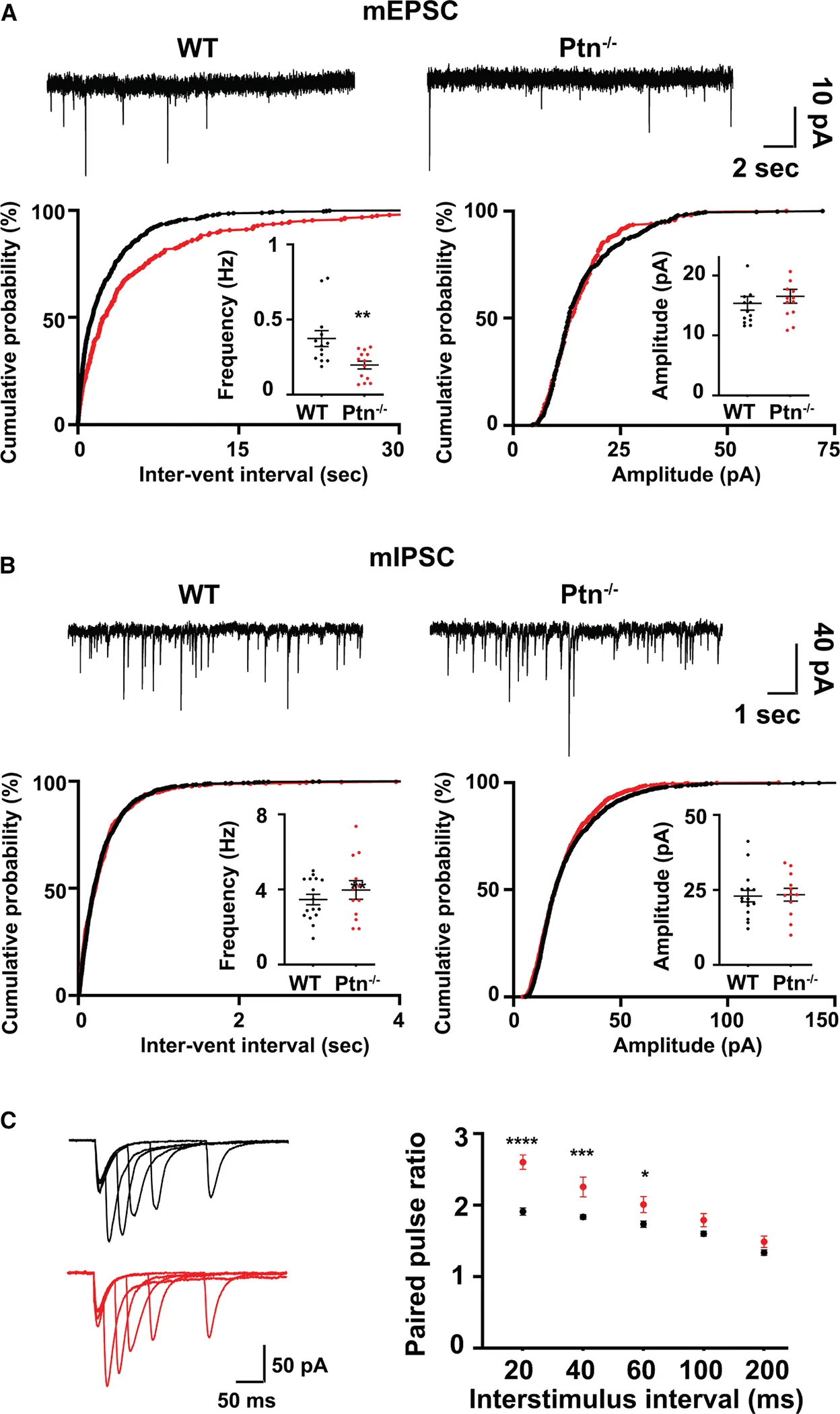
Ptn regulates excitatory synaptic transmission *in vivo* (A) Representative traces and cumulative distributions of mEPSCs recorded from CA1 neurons in WT and Ptn^−/−^ mice. mEPSC frequency was reduced without changes in amplitude. Unpaired *t* test; **p < 0.01; n = 18 cells from 5 WT mice and 12 cells from 4 Ptn^−/−^ mice. (B) mIPSC recordings show no significant changes in inhibitory synaptic transmission. n = 16 cells from 4 WT mice and 12 cells from 3 Ptn^−/−^ mice. (C) Paired-pulse ratios were elevated in Ptn^−/−^ mice. Two-way ANOVA with Bonferroni’s test, genotype effect; *p* < 0.0001, *p < 0.05, ***p < 0.001, and ****p < 0.0001; n = 17 cells from 5 WT mice and 14 from 3 Ptn^−/−^ mice. Error bars, SEM.

Given the deficits in RIM clustering in Ptn^−/−^ mice, we assessed synaptic function using whole-cell voltage-clamp recordings from CA1 pyramidal neurons in acute hippocampal slices. We found reduced frequency, but not amplitude, of miniature excitatory postsynaptic currents (mEPSCs) in Ptn^−/−^ neurons (Figure 6A). In contrast, miniature inhibitory postsynaptic currents (mIPSCs) were not affected (Figure 6B), indicating a selective role for Ptn in excitatory synaptic transmission in CA1 neurons. The reduction in mEPSC frequency, despite normal synapse density, suggested reduced presynaptic release probability. To test this, we measured the paired-pulse ratio (PPR) of evoked transmission at CA3-CA1 synapses that is inversely correlated with the initial release probability.^33^ Ptn^−/−^ mice exhibited an increased PPR compared with WT mice (Figure 6C). Together with reduced RIM clustering observed *in vivo*, these results indicate that Ptn contributes to presynaptic assembly and efficient neurotransmitter release.

### Ptn^−/−^ mice exhibit impaired contextual fear discrimination

To determine whether the synaptic deficits observed in Ptn^−/−^ mice were associated with behavioral alterations, we selected a focused set of assays. Open-field and elevated plus maze tests were used to evaluate locomotor activity and anxiety-like behavior, whereas a contextual fear discrimination task was selected based on its reported dependence on CA3-CA1 synaptic function.^34^ Together, these assays provide a targeted assessment of the behavioral domains relevant to the synaptic phenotypes observed in this study.

**Figure 7.**
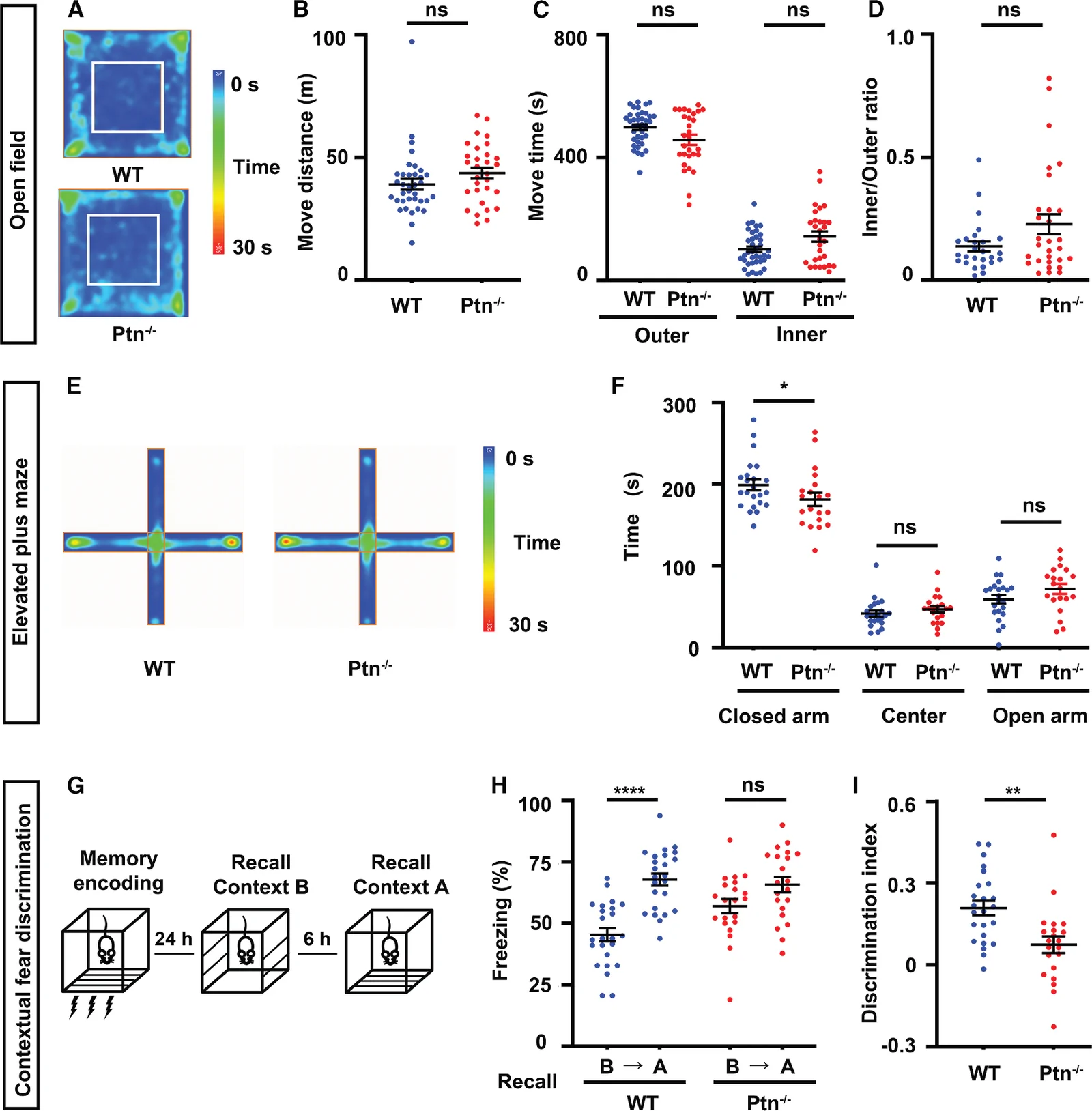
Ptn^−/−^ mice show impaired contextual discrimination (A–D) Open-field analysis showing no differences in locomotion or spatial preference between the WT and Ptn^−/−^ mice. Distance traveled (B), time spent in inner versus outer areas (C), and inner/outer ratio (D) were unchanged. Three-way ANOVA: no sex, genotype, or position effect. Pooled analysis, Welch’s *t* test (B), two-way ANOVA (C) with Bonferroni’s test or Mann-Whitney test (D). n = 19 male + 17 female WT mice and 13 male + 17 female Ptn^−/−^ mice. (E and F) Elevated plus maze analysis. Ptn^−/−^ mice show a modest but statistically significant reduction in time spent in closed arms (F). Three-way ANOVA: no sex, genotype, or arm position effect; *p* < 0.0001. Pooled analysis, two-way ANOVA with Bonferroni’s test; *p < 0.05. n = 10 male + 13 female WT mice and 9 male +11 female Ptn^−/−^ mice. (G) Schematic of the contextual fear discrimination assay. (H and I) Ptn^−/−^ mice showed impaired discrimination between contexts A and B, measured by freezing levels (H) and discrimination index (I). Three-way ANOVA: sex effect, *p* = 0.14; genotype effect, *p* = 0.036; context effect, *p* < 0.0001. Pooled analysis: two-way ANOVA with Bonferroni’s test; ****p < 0.0001 in (H) or Welch’s *t* test (**p < 0.01) in (I). n = 12 male + 12 female WT mice and 8 male + 11 female Ptn^−/−^ mice. Error bars, SEM.

In the open-field test, Ptn^−/−^ mice showed normal locomotor activity, including total distance traveled and time spent in the inner and outer zones (Figures 7A–7C). The inner/outer ratio did not differ significantly between genotypes (Figure 7D), suggesting no detectable difference in anxiety-like behavior under these conditions. In the elevated plus maze, Ptn^−/−^ mice spent slightly less time in closed arms, without a significant increase in time spent in open arms (Figures 7E and 7F). Together, these findings do not support a consistent alteration in anxiety-like behavior between Ptn^−/−^ and control mice.

In the contextual fear discrimination task (Figure 7G), WT mice successfully discriminated between contexts, exhibiting higher freezing in the shock-associated context (context A) than in the neutral context (context B) (Figures 7H and 7I). In contrast, Ptn^−/−^ mice failed to distinguish between the two contexts, showing similar levels of freezing in both environments. Notably, freezing levels in the shock-associated context (context A) were comparable between the WT and Ptn^−/−^ mice, indicating preserved fear memory responses. Together, these findings indicate impaired contextual discrimination in Ptn^−/−^ mice, consistent with synaptic deficits observed at CA3-CA1 synapses.

## DISCUSSION

Despite the association of Ptn with diverse neurodevelopmental and neurodegenerative disorders, its physiological function in the brain has remained poorly understood. Here, we identify HS-modified Nrxn1 as a prominent synaptic receptor for Ptn in the mouse brain and uncover a role of Ptn in presynaptic organization, neurotransmitter release, and hippocampus-dependent contextual discrimination. Together, our findings support a model in which molecular information encoded within extracellular glycans contributes to the organization and function of presynaptic machinery in neural circuits.

### Glycan-mediated Ptn interaction with Nrxn1 in the mouse brain

While Ptn functions have largely been attributed to the receptor Ptprz1, our proteomic and biochemical analyses identify Nrxn1 as another major endogenous Ptn-binding partner in the brain. Importantly, the Ptn-Nrxn1 interaction in the brain requires HS modification of Nrxn1, supporting a glycan-dependent molecular recognition mechanism *in vivo*. Our results demonstrated that HS modification of Nrxn1 is required for its robust association with Ptn in the brain. This conclusion is strengthened by genetic disruption of the Nrxn1 HS attachment site rather than enzymatic HS removal alone. Glycan microarray analyses further demonstrated that Ptn recognizes defined HS sulfation motifs, with binding governed by precise sulfation patterning, rather than the overall sulfate density or negative charge.

At the same time, HS structure alone is unlikely to fully account for this selectivity. Our data indicate that Ptn preferentially associates with Nrxn1 over GPC4 and other abundant HSPGs in the brain (Figure 2D; Figure S2). Several non-mutually exclusive mechanisms may contribute to this preferential association. For example, distinct Ptn-binding HS motifs may be preferentially associated with Nrxn1. Consistent with this model, HS chains exhibit substantial structural heterogeneity, even within a single cell, and emerging evidence suggests that HSPG core proteins can influence HS structure and presentation.^35^ Indeed, distinct HS epitopes form spatially segregated clusters on the cell surface and are attached to different glypican family members within the same cell,^35^ raising the possibility that HSPG core proteins contribute to the generation and spatial presentation of distinct HS-binding platforms. However, the spatiotemporal distribution of specific HS sulfation motifs *in vivo*, the proteoglycans to which they are attached, and their localization within defined neural cell types or synaptic compartments remain poorly understood owing, in large part, to the current technological limitations.

In addition to HS structural features, the Nrxn1 core protein itself may further contribute to the specificity of the Ptn-Nrxn1 interaction. Differences in Ptn binding among Nrxn isoforms raise the possibility that the protein context may influence the selective association of Ptn with HS-modified Nrxn1. Moreover, the spatial proximity of Ptn and Nrxn1 *in vivo* may also favor their interaction. Indeed, both proteins are expressed in neurons and astrocytes,^19,36^ raising the possibility that cell-type-specific expression and local secretion of Ptn contribute to its preferential association with Nrxn1 within specific neural microenvironments. Together, these findings suggest that both HS structural features and protein context contribute to the association of Ptn with Nrxn1.

### Ptn-HS-Nrxn pathway and interpretation of mechanism

At the functional level, we show that both HS and Nrxns are required for Ptn-induced presynaptic assembly in cultured neurons. Although these findings support a model in which Ptn acts through HS-modified Nrxns, the HS-Nrxn interactions are known to mediate multiple extracellular signaling pathways.^5,27,28,37^ Therefore, perturbation of Nrxn HS is expected to affect multiple ligands, and the contribution of Ptn likely represents one component of broader HS-Nrxn-dependent signaling mechanisms.

Comparison of *in vivo* phenotypes further supports this interpretation. Disruption of HS modification on Nrxn1 caused broad synaptic defects, including impairments in both presynaptic and postsynaptic structures and functions.^5^ In contrast, Ptn^−/−^ mice exhibited more selective alterations, primarily affecting presynaptic organization and release probability, without detectable changes in synapse density or postsynaptic spine structure (Figures 5 and 6; Figure S5). These differences suggest that HS-modified Nrxns function as a signaling platform that integrates multiple extracellular cues, with Ptn representing a presynaptic regulatory component of this pathway.

Notably, an independent Ptn^−/−^ mouse line has been reported to exhibit reduced cortical spine density in adulthood,^19^ suggesting that Ptn-dependent functions may vary across brain regions and developmental stages. In addition to Nrxn1, our proteomic analyses identified other candidate Ptn-interacting proteins, including PTPσ, PTPδ, and Gpm6a (Table S1). These findings suggest that Ptn may engage a broader network of synaptic proteins beyond Nrxns. Consistent with this possibility, we found the formation of the Ptn-PTPσ complex, which partially depends on Nrxn1 HS (Figure S1C), raising the possibility that Nrxn1-associated HS may facilitate interactions between Ptn and additional synaptic components. Such context-dependent interactions may contribute to the diverse phenotypes associated with Ptn deletion. Further studies are required to define the functional roles of these candidate interactions.

### Comparison with other HS-binding synaptic organizers

Several synaptic organizing proteins, including LRRTM4 and GPR158, bind to HSPGs to regulate synapse development.^7–9,27,38^ However, Ptn exhibits different receptor preferences and functional outcomes. GPR158 engages GPC4 at CA3 thorny excrescence synapses, whereas LRRTM4 interacts with both GPCs and Nrxns at dentate gyrus synapses.^7–9,38^ In contrast, our data suggest that Nrxn1 is a prominent Ptn-associated HSPG in the hippocampus.

This molecular divergence aligns with their functional impacts on synapse development. LRRTM4 deletion reduces synapse density and AMPAR levels without affecting presynaptic release, whereas GPR158 deletion impairs synaptic morphology and function but enhances transmitter release.^7,8^ In contrast, Ptn regulates presynaptic development and function. Together, these results suggest that HS-binding synaptic organizers may act through distinct HSPGs to shape different aspects of synaptic properties.

### Functional consequences on behavior

At the behavioral level, Ptn^−/−^ mice exhibited reduced contextual discrimination, without evidence of increased anxiety-like behavior or locomotor deficits. Notably, freezing behavior in the shock-associated context is preserved, whereas increased freezing in the neutral context reflects impaired discrimination between similar environments. As normal CA3-CA1 synaptic function is required for proper performance in this contextual discrimination paradigm,^34^ the behavioral alterations observed in Ptn^−/−^ mice are consistent with the presynaptic deficits observed at CA3-CA1 synapses.

### Implications and broader significance

Nrxn1 mediates a central pathway in synapse development that is strongly linked to a range of neurodevelopmental and neurodegenerative disorders.^39,40^ Alterations in HS biosynthesis pathways have also been associated with similar conditions.^41–43^ Ptn has been linked to a range of neurological and psychiatric disorders, including neurodevelopmental syndromes and neurodegenerative diseases,^11,14,15,17,19,20,22^ although its physiological function in the brain has remained obscure. By identifying Nrxn1 as an endogenous binding partner of Ptn and revealing a role for Ptn in presynaptic development, our work provides a mechanistic framework linking HS-dependent signaling to synaptic mechanisms important for proper brain function.

More broadly, our results support a model in which HSPGs function not merely as non-specific electrostatic scaffolds but as selective extracellular recognition interfaces. These findings raise the possibility that additional secreted factors may engage HS-modified Nrxns to regulate synapse development. Several secreted synaptic organizers, including FGFs, Wnts, and thrombospondins, are known to interact with HS *in vitro*.^44^ Determining whether such factors similarly engage HS-modified Nrxns or other HSPGs *in vivo* is an important direction for future work.

### Limitations of the study

Several limitations of this study should be noted. First, although our data support the role of HS-modified Nrxn1 in mediating Ptn function, we did not directly test whether HS modification on Nrxn1 is required for Ptn-dependent presynaptic assembly *in vivo*, as the perturbation of Nrxn1 HS would affect interactions with multiple ligands and complicate interpretation of Ptn-specific effects. Second, while glycan microarray analyses provide insight into Ptn HS binding preferences, the extent to which these motifs and other motifs like 3-*O*-sulfation are present on endogenous brain HSPGs *in vivo* remains unclear. Addressing this question requires the development and integration of cell type-specific approaches and *in vivo* glycomic analyses, which remain technically challenging. Third, the cellular sources of Ptn in the brain and their contributions to synapse development remain to be determined. Because Ptn is a secreted protein expressed by multiple cell types in the brain, including neurons and glia, the global Ptn knockout used here does not distinguish between the cellular origins of Ptn or the specific sites at which it acts. Given its secreted nature, Ptn may function as a locally acting extracellular signal within neural circuits. Future studies using cell type-specific manipulations are, therefore, important to define the cellular sources, sites of action, and circuit-specific functions of Ptn during synapse development.

## RESOURCE AVAILABILITY

### Lead contact

Requests for further information and reagents may be directed to and will be fulfilled by the lead contact, Peng Zhang (pxz187@case.edu).

### Materials availability

This study did not generate new unique reagents.

### Data and code availability

- **Data:** The mass spectrometry proteomics data have been deposited to the ProteomeXchange Consortium via the PRIDE^45^ partner repository and are publicly available under accession number PXD081065. The accession number is also listed in the key resources table.
- **Code:** This paper does not report original code.
- **All other items:** Any additional information required to reanalyze the data reported in this paper is available from the lead contact upon request.

## STAR★METHODS

### EXPERIMENTAL MODEL AND STUDY PARTICIPANT DETAILS

#### Transgenic mice

The Nrxn1ΔHS knock-in mouse line was described previously.^5^ The Ptn knockout mouse line was originally generated by the Dr. Muramatsu group^46^ and was shared by Dr. Polyxeni Philippidou at Case Western Reserve University. All mice were housed and bred in the Animal Resource Center of Case Western Reserve University School of Medicine on a 12h/12h, light/dark cycle with *ad libitum* access to food and water. All procedures were approved by the Institutional Animal Care and Use Committees (IACUC) at Case Western Reserve University.

#### Cell culture

Primary rat hippocampal neuron cultures were prepared from embryonic day 18 rat embryos as described previously.^5^ Both sexes were pooled and used. In brief, 250,000 hippocampal neurons were seeded in a 60 mm culture dish, in which 5 coverslips with wax dots were cultured with a glial feeder layer. The neurons were cultured in a neural basal medium + B27 + glutamate-Max. At 2 days *in vitro* (DIV), cytosine arabinoside (5 μM) was added to prevent overgrowth of glial cells.

HEK293FT cells were cultured in DMEM media supplemented by 10% or 3% fetal bovine serum.

### METHOD DETAILS

#### Preparation of crude membrane fraction and synaptic plasma membranes (SPM)

Mouse brains were rapidly removed and rinsed with cold buffer A (320mM sucrose, 4 mM HEPES-NaOH, pH7.4). All buffers included protease inhibitor cocktail (PIC; Roche Cat# 05056489001) throughout the procedure. The cortex and hippocampus were dissected out quickly and pooled. The tissues were homogenized in 1:10 (weight/volume) buffer A using a Teflon homogenizer (10 strokes, manually). While a small fraction of this homogenate was collected for blots as lysate input, the remaining was centrifuged for 10 min at 1000g to remove nuclei. The supernatant S1 was centrifuged for 15 min at 12,500 g. The pellet P2 was resuspended in cold buffer A and pelleted again at 12,500 g. The resulting pellets P2′ were referred to as the crude membrane fraction and stored at −80°C.

To prepare the SPM, P2′ pellets were homogenized in ice-cold 4 mM HEPES buffer pH7.4 and rotated for 25 min in the cold room for complete lysis. The lysates were centrifuged at 25,000 g for 25 min. The pellets were resuspended with buffer A and loaded in a sucrose gradient buffer (41%, 34%, and 27%) for the centrifugation (~221,600 g) for 2 h. The white materials between the 34% and 41% sucrose layers were collected, diluted to 0.32 M sucrose, and pelleted to obtain the SPM (200,000 g, 30 min).

#### Pull-down experiments with Ptn-hFc and hFc

30 μg of Ptn-hFc or 30 μg hFc proteins were incubated with 90 μL Protein-A agarose beads for 4 h in the cold room in 1 mL Tris buffer [50 mM Tris^.^HCl, pH 7.4, 150 mM NaCl], followed by washing three times with washing buffer [50 mM Tris.HCl, pH 7.4, 150 mM NaCl, 0.1% DMM +0.01% CHS (Anatrace, D310-CH210), and PIC]. The agarose beads with bound hFc proteins were mixed with 6 mg of lysates of crude membrane fractions made from hippocampus and cortex of three C57BL/6J mouse brains [lysis buffer: 50 mM Tris.HCl, pH 7.4, 150 mM NaCl, 1% DMM +0.1% CHS (Anatrace, D310-CH210), and PIC] overnight in the cold room. Beads were washed three times with washing buffer. Bound proteins were eluted in 1*x* Laemmli Sample Buffer and resolved in a 4–20% gradient gel. The gel was submitted to the Proteomics & Metabolomics Core at Cleveland Clinic for MS proteomics analysis. For the validation of MS results, a small amount of Ptn-hFc and hFc proteins was used in the same procedure and proteins in gels were transferred to PVDF membranes for western-blot.

#### LC-MS/MS to identify Ptn-interacting proteins in the brain

LC–MS/MS analysis was performed at the Proteomics Core Facility at the Cleveland Clinic. Membrane fractions from mouse cortex and hippocampus were subjected to SDS-PAGE, and each lane was divided into 12 gel bands. Gel pieces were washed and destained in 50% ethanol and 5% acetic acid, followed by dehydration in acetonitrile. Proteins were reduced with dithiothreitol (DTT) and alkylated with iodoacetamide prior to in-gel digestion.

Proteins were digested overnight at room temperature with sequencing-grade trypsin (5 ng/μL in 50 mM ammonium bicarbonate). Peptides were extracted from gel pieces using two sequential incubations with 30 μL of 50% acetonitrile containing 5% formic acid. Extracts were combined, concentrated to <10 μL using a SpeedVac, and resuspended in 0.1% formic acid to a final volume of ~30 μL for LC–MS analysis.

LC–MS/MS analysis was performed on a Bruker timsTOF Pro2 mass spectrometer (Bruker Daltonik GmbH, Bremen, Germany) operating in positive ion mode and coupled to a CaptiveSpray ion source. Peptides were separated on a 15 cm × 75 μm inner diameter C18 reversed-phase column (ReproSil AQ, 1.9 μm, 120 Å ). Samples (1 μL) were injected and separated using an acetonitrile/ 0.1% formic acid gradient at a flow rate of 0.3 μL/min and introduced online into the mass spectrometer.

Data were acquired in data-dependent acquisition (DDA) mode using parallel accumulation–serial fragmentation (PASEF). Each acquisition cycle consisted of one TIMS-MS survey scan followed by 10 PASEF MS/MS scans, with a total cycle time of 1.2 s. The TIMS-MS survey scan covered an ion mobility range of 0.60–1.6 Vs/cm^2^ and an m/z range of 100–1,700, with a ramp time of 166 ms. MS/MS spectra were acquired with collision energies ranging from 20 eV (0.6 Vs/cm^2^) to 59 eV (1.6 Vs/cm^2^). Precursor ions with charge states of 2–5 were selected using a target intensity of 20,000 a.u. and an intensity threshold of 2,500 a.u. Dynamic exclusion was applied for 0.4 s.

Raw data were processed using PEAKS Online and searched against the mouse UniProt/SwissProt database. Search parameters included trypsin specificity with up to two missed cleavages, carbamidomethylation of cysteine as a fixed modification, and methionine oxidation as a variable modification. Peptide and protein identifications were filtered at a false discovery rate (FDR) of ~1%, with a minimum of two peptides required per protein.

Protein quantification was performed using both spectral counts and label-free intensity-based measurements (LFQ). Enrichment of candidate binding partners was assessed relative to hFc control samples based on LFQ intensity ratios, and proteins showing ≥2-fold enrichment were prioritized for further analysis.

#### Immunoprecipitation from lysates of mouse brains

The crude membrane fractions were lysed in the lysis buffer [50 mM Tris.HCl, pH 7.4, 150 mM NaCl, 1% DMM +0.1% CHS, with PIC] for 2 h in the cold room. The lysates were centrifuged at 17,000 g for 10 min at 4°C. 10 μg HS20 antibodies or anti-hFc control antibodies were incubated with 20 μL Protein-A agarose overnight in the cold room. The bound antibodies and beads complex were then incubated with 2 mg of solubilized proteins overnight in the cold room. The beads were then washed three times with wash buffer (50 mM Tris.HCl, pH 7.4 + 150 mM NaCl, 0.1% DMM +0.01% CHS). Bound proteins were eluted with Laemmli sample buffer at 65°C for 10 min and separated by running SDS-PAGE gels.

#### Western blot

PVDF membranes were blocked by 5% skim-milk dissolved in 1×TBS buffer (150 mM NaCl, Tris.HCl pH 7.4) for 1 h at room temperature. Membranes were rotated with primary antibodies in 1×TBS buffer containing 3% BSA and 0.05% Tween 20 overnight in the cold room, and secondary antibodies in the same buffer for 40 min at room temperature. Membranes were washed by 1xTBS containing 0.05% Tween 20 and visualized by Immobilon Chemiluminescent HRP substrate kit (Millipore, Cat# WBKLS0500) under ChemiDoc imaging system (Bio-Rad). Primary antibodies: anti-neurexin-1 antibody (1:1000), anti-pan-neurexins (1:2000), anti-PTPσ (1:600), anti-GPC4 (1:3000), anti-Ptn (1:2000), anti-HS stub (3G10, 1:3000), anti-PSD95 (1:500), anti-β-actin (1:5000).

#### HS microarray binding assay

Glycan microarrays were generated using chemically-synthesized HS tetrasaccharides and used to assay protein binding as described.^26^ Briefly, the microarray slides were blocked in 10% w/v BSA in TBST (50 mM Tris pH 7.5, 150 mM NaCl, 0.1% Tween 20) for 1 h at room temperature with shaking (200 rpm). Ptn-hFc or LRRTM4-hFc was then added to the slide at a concentration of 1 μg/mL in 1% w/v BSA in TBST for 1 h at room temperature with shaking. Slides were then washed with TBST twice for 5 min with shaking. Goat anti-human IgG Fcγ, Alexa 647 was added to the slides at a concentration of 1 μg/mL in 1% w/v BSA in TBST for 1 h at room temperature with shaking. The slides were protected from light starting at this point. After washing with TBST twice for 5 min each, the slides were washed with PBS twice for 5 min with shaking, then dipped twice in ddH2O and gently dried with 0.2 micron- filtered air. Fluorescence was detected using an Agilent G2565CA Microarray Scanner at 647 nm. Quantification of fluorescence was performed using ImageJ, and results were plotted with Prism 10 software. All experiments were performed in duplicate. Sulfation logos were computationally generated from the microarray data as reported previously.^26^

#### Ptn-hFc binding to Nrxns in primary rat hippocampal neurons

Dissociated primary rat hippocampal neurons (1 × 10^6^ cells) were transfected with pLL3.7-hSyn-V5-Nrxn1α, V5-Nrxn1α-ΔHS, YFP-P2A-V5-Nrxn2α, YFP-P2A-V5-Nrxn2α-ΔHS, or V5-Nrxn3α constructs (all Nrxnα isoforms lacked splice sites 4 and 5) and plated onto coverslips. Constructs were generated by Gibson assembly, and all cDNA sequences were verified by Sanger sequencing. At DIV3, recombinant Ptn-hFc proteins were added to live neurons in conditioned culture medium and incubated for 1 h under chilled conditions to minimize protein internalization. Neurons were then washed extensively with ice-cold PBS to remove unbound proteins and fixed with 4% paraformaldehyde and 4% sucrose in PBS (pH 7.4) for 12 min at room temperature, followed by three washes with PBS.

After blocking, neurons were incubated with mouse anti-V5 antibodies followed by goat anti-mouse IgG2a-Alexa Fluor 568 to detect V5-Nrxns and goat anti-human Fc-Alexa Fluor 647 to visualize bound Ptn-hFc proteins.

#### Ptn-hFc beads co-culture with primary rat hippocampal neurons

Ptn-hFc or hFc proteins were mixed with goat anti-human IgG magnetic beads (Spherotech # HMS-40–10) in PBS/BSA (100 μg/mL) buffer for 2 h at room temperature. After washing out unbound proteins, the Ptn-hFc or hFc conjugated beads were added to DIV13 neurons grown on coverslips for 45 min in a 12-well plate before putting the beads-neuron co-culture coverslips back to home glia dishes. The co-culture continued for 20–24 h in a CO_2_ incubator before fixation. Neurons were treated by heparinases I, II, and III (0.5 U/mL) or mock control in glial conditioned medium for 2 h in a 37°C CO_2_ incubator. For knocking down neurexins, AAV-rNrx-TKD or AAV-shMorB were incubated with DIV3 neurons on coverslips for 4 h in a 12-well plate. After incubation, coverslips were returned to home glial dishes to grow until the beads-neuron co-culture experiments.

The beads-neuron co-culture were fixed by 4% PFA, 4% sucrose, 1*×* PBS (pH7.4) for 12 min at room temperature, followed by three washes with 1*×* PBS and one wash of 1*×* PBS with 0.02% Triton X-100. After blocking, primary and secondary antibodies were performed as previously reported.^5^ Primary antibodies include anti-synapsin1 (1:20000), anti-RIM1/2 (1:2000), anti-MAP2, anti-pan-neurexins (1:2000), and anti-vGlut1 (1:10000). We used highly cross-adsorbed, Alexa dye conjugated secondary antibodies generated in goat toward the appropriate species and isotypes (1:1000; ThermoFisher; Alexa 488, Alexa 568, and Alexa 647 labeled secondary antibodies). AMCA conjugated anti-chicken IgY (donkey IgG; 1:400; Jackson ImmunoResearch; 703–155-155) was used for visualizing dendrites.

Sets of cells were stained simultaneously and imaged with identical settings. Images were collected on a Zeiss AxioImager M2 microscope with 63*×*/1.4 NA or 40*×*/1.3 NA oil objectives, a Hamamatsu Orca-Flash4.0 CMOS camera, and custom filters. For quantification in ImageJ2, integrated intensity of presynaptic proteins was normalized to the area of beads without MAP2.

#### AAV production and purification

The procedure was adapted from the previous study.^47^ pAAV-rNrx-TKD or pAAV-shMorB was cotransfected with pUCmini-iCAP-PHP-eB (Addgene #103005) and pHelper (gift from Lin Mei, originally from Agilent) plasmids in a ratio of 1:4:2 into HEK293FT cells. The second day, media were changed with DMEM supplemented with 3% fetal bovine serum for 72 h. Conditioned media were collected and precipitated with 1/5 (v/v) 40% PEG8000 overnight. Meanwhile, the cells were collected in PBS and extracted by 10% chloroform followed by high salt buffer and PEG8000 precipitation. The pellets from both conditioned medium and cells were digested by Benzonase Nuclease in SAN buffer (50 mM Tris-HCl, pH 8.0, 150 mM NaCl, 2 mM MgCl_2_), followed by iodixanol density gradient centrifugation (15%, 25%, 40%, and 60% weight/volume). The virus was harvested from the 40/60% interface and the 40% layer. The virus was diluted with 1xPBS three times for buffer change and concentrated in an Amicon Ultra-15 centrifugal filter device.

#### Dil label for spine density

The procedure was adapted from the previous study.^48^ 6 weeks-old mice were fixed with 4% PFA by transcardial perfusion with post fixation overnight at 4°C. 150 μm coronal sections were collected using vibratome. Solid DiI crystals (catalog # D-282 invitrogen) were vortexed into fine crystals and applied to the slices using a sharp borosilicate glass micropipette under a dissecting microscope with gentle poking. The slices were kept at 4°C for 24–48 h to allow the dye to diffuse along the neuronal membrane. Then, the slices were mounted onto coverslips with mounting solution (Polyvinyl alcohol 13.3%, 0.067M Tris.HCl pH 8.5, glycerol 66.7%, DABCO 2.5%) for imaging. 3D Images were collected by Zeiss LSM800 Ariyscan with a 63*x* oil objective (NA = 1.4). The genotypes were blinded for data analysis. Maximum intensity projection from 3D images was used for manual counting for spine density.

#### MAP procedure

The MAP procedure is similar to our previous publication^31^ with additional considerations. First, all samples from both genotypes were processed in the same container during the chemical incubation, gelation, and immunolabeling steps. Second, we withdrew the MAP procedure for analysis if expansion factors differ more than 5% between hippocampal slices. This predetermined criterion ensures a consistent and fair comparison among brain slices with different genotypes. Primary antibodies in this study included PSD95 (1:200) and RIM1/2 (1:250). Images were collected on a Zeiss LSM800 confocal microscope with the Ariyscan function, a water 63*×* objective (Na = 1.2), pixel size (x, y, z) = 40, 40, 220 nM. Images were blinded for genotype information and analyzed by Zeiss Arivis.

#### Hippocampal slice-electrophysiology

Electrophysiology recording from CA1 region of mouse brain was performed as previously described.^49^ Briefly, isoflurane-anesthetized P15-P18 mice were decapitated, and brains were quickly dissected out. The brains were subsequently sectioned into 400 μm coronal slices in ice-cold artificial cerebrospinal fluid (ACSF) balanced with 95% O_2_ and 5% CO_2_ using a vibratome (VT1200S Leica). The composition of ACSF was as follows (in mM): 125 NaCl, 2.5 KCl, 2 CaCl_2_, 2 MgCl_2_, 1.25 NaH_2_PO_4_, 26 NaCO_3_, 25 glucose (pH 7.35 310–320 mOsm). The coronal slices in ACSF were incubated at 31°C for 30 min and then transferred to room temperature to recover for 60 min before recording.

Brain slices were transferred into the recording chamber with continuous perfusion of 95% O_2_ and 5% CO_2_ balanced ACSF at 2 mL/min. Whole cell patch was performed with glass patch pipettes (resistances ranging between 3 and 6 MΩ) pulled with borosilicate glass capillary tubes (World Precision Instruments) using a puller (P-97, Sutter Instruments).

For miniature excitatory postsynaptic currents (mEPSCs) recording, cells were holding at −70 mV and recording for 2 min in the presence of 1 μM tetrodotoxin (Abcam) and 10 μM bicuculline methiodide (Abcam). The internal solution contained (in mM): CsMeSO_3_ 130, NaCl 8, MgCl_2_ 2, HEPES 10, EGTA 0.5, ATP 4, and QX-314 5 (pH 7.2). For recording of miniature inhibitory postsynaptic currents (mIPSCs), the internal solution contained (in mM): CsCl 140, CaCl_2_ 0.1, MgCl_2_ 2, HEPES 10, BAPTA 10, ATP 4, and QX314 5 (pH 7.2). Meantime, 1 μM of tetrodotoxin (Abcam), 10 μM of bicuculline methiodide (Abcam) and 20 μM of CNQX (Abcam) were added in the ACSF. The same internal solution was used for PPR recording, while the brain slices were stimulated with glass pipettes filled with 1M NaCl and positioned within ~100–200 μm from the patched cell in CA1 *stratum radiatum*. The PPR was recorded with whole cell voltage clamp holding at −70 mV, while two brief, sequential stimuli were delivered at varying interstimulus intervals. Signals were amplified (Axopatch 200B, Axon Instruments), filtered at 2 kHz and digitized at 10 kHz. Series resistance (Rs) was compensated (40–50%). Cell with consistent Rs were included for data analysis.

#### Mouse behaviors

All behavioral studies were done in the Case Western Reserve University mouse behavior phenotype core with researchers blind to genotype. Littermates from heterozygous parents were used to control genetic backgrounds in all behaviors. Both male and female mice age 10- to 18-weeks-old were used in behavioral tests. Mice were handled for at least a week prior to testing. Before testing, mice were habituated to the behavior room for 30–60 min. Anymaze software was used to record and track mice behaviors. Data were analyzed using Prism.

##### Open-field test

The procedure was adapted from the previous study.^50^ The open field maze consisted of four 49.5 cm (length) × 49.5 cm (width) × 38 cm (height) active chambers. The chambers were extensively cleaned with 30% ethanol to remove any scent between tests. Mice were placed in the center of the chamber and allowed to explore the chamber for 10 min. A 29.5 cm (length) × 29.5 cm (width) area in the middle of the of the chamber was defined as the center zone, and the 29.5 cm (length) × 10 cm (width) area alongside the periphery area was defined as the outer zone or periphery zone.

##### Elevated plus maze

The procedure was adapted from the previous study.^51^ The Elevated Plus Maze consisted of four perpendicular arms with two open without walls and two enclosed by 19 cm high walls. Each arm was 35 cm (length) × 6.35 cm (width) and the center square at the crossing was 6.35 cm (length) × 6.35 cm (width). The whole apparatus was 75 cm off the floor. Mice were placed in the center square facing one of the closed arms at the start of the experiments and explored the arms for 5 min.

##### Contextual fear discrimination

The procedure was adapted from the previous study^52^ On day one, mice were introduced to the fear conditioning chamber with metal bars on the bottom and silver metal walls named as context A (Med Associates Video Fear S.A.C NIR-022MD) for 3 min followed by a 2-s 0.50 mA footshock. After the footshocks, mice were returned to their home cage. Twenty-four h after the footshock, mice were placed back into the fear conditioning chamber with substantial modification named context B (a soft white plastic pad covering the metal bar and chamber walls were covered with green and white striped paper. Mice were allowed to explore in context B for 3 min 6 h later, mice were placed in context A and allowed to explore for 3 min. The freezing behavior, defined as complete suppression of movement except for those associated with respiration, were counted in each context using the freezing count software. The discrimination ratio was determined as (freezing time in A − freezing time in B)/(freezing time in A + freezing time in B).

### QUANTIFICATION AND STATISTICAL ANALYSIS

Statistical analyses were performed using GraphPad Prism 10. The statistical tests used for individual experiments are specified in the corresponding figure legends. For comparisons between two groups, two-tailed unpaired Student’s t tests or Welch’s t tests were used for normally distributed data, as appropriate. Mann–Whitney tests were used when the assumptions of parametric testing were not met. Comparisons involving more than two groups were performed using one-way ANOVA followed by Bonferroni’s multiple-comparisons tests. Two-way or three-way ANOVA followed by Bonferroni’s multiple-comparisons tests was used to evaluate experiments involving multiple factors, including genotype, sex, behavioral context, arm position, or interstimulus interval, as indicated in the figure legends. Unless otherwise stated, data are presented as mean ± SEM. Glycan microarray data are presented as mean ± SD.

Exact sample sizes and the definitions of n are provided in the corresponding figure legends. For biochemical experiments, n represents independent experiments; for cultured-neuron imaging experiments, n represents individual cells obtained across the indicated number of independent cultures; for electrophysiological experiments, n represents recorded cells, with the number of mice also reported; for behavioral experiments, n represents individual mice; and for expansion-microscopy experiments, n represents individual mice. For the expansion-microscopy analysis in Figure 5, individual synapses are displayed to illustrate their distributions but were not treated as independent biological replicates; statistical comparisons were performed using mice as the biological replicates. Statistical significance was defined as *p* < 0.05.

## Supplementary Material

1

Supplemental information can be found online at https://doi.org/10.1016/j.celrep.2026.117827.

## Figures and Tables

**KEY RESOURCES TABLE:** 

REAGENT or RESOURCE	SOURCE	IDENTIFIER

Antibodies		

Mouse anti-V5	ThermoFisher	Cat# R960CUS, RRID:AB_2792973
Rabbit anti-pan-Nrxn	Millipore	Cat#ABN161; RRID: AB_10917110
Mouse anti-HS stub (3G10)	AMSBIO LLC	Cat# 370260; RRID: AB_10892311
Rabbit anti-β Actin	Abcam	Cat#ab8227; RRID: AB_2305186
HS20 (anti-HS)	InVivoMAb	Cat#BE0412; RRID: AB_3696136
Rabbit anti-Nrxn1	Synaptic Systems	Cat# 175103; RRID:AB_10697816
Rabbit anti-PTPσ	Proteintech	Cat# 13008-1-AP; RRID:AB_10858319
Rabbit anti-GPC4	Proteintech	Cat# 13048-1-AP; RRID:AB_10640157
Mouse anti-PSD95	NeuroMab	Cat# K28/43; RRID:AB_2877189
Rabbit anti-Ptn	Thermo Scientific	Cat# PA5-94984; RRID:AB_2806790
Anti-RIM	Synaptic Systems	Cat# 140205; RRID:AB_2631216
Anti-Synapsin1	Synaptic Systems	Cat# 106011; RRID:AB_2619772
Chicken anti-MAP2	Synaptic Systems	Cat# 188006; RRID:AB_2619881
Rabbit anti-vGlut1	Millipore	Cat# AB5905; RRID:AB_2301751

Bacterial and virus strains		

AAV-PHP-eB-shMorB	This paper	N/A
AAV-PHP-eB-rNrx-TKD	This paper	N/A

Chemicals, peptides, and recombinant proteins		

CNQX	Abcam	Cat#ab120044
DL-APV	Abcam	Cat#ab120271
Tetrodotoxin (TTX)	Abcam	Cat#ab120054
SR95531 hydrobromide (Gabazine)	Tocris	Cat#1262
(R)-CPP	Tocris	Cat#0247
Tetrodotoxin (TTX)	Tocris	Cat#1069
PTN-hFc	Sino Bio	Cat# 5100-M01H
Human IgG Fc	Thermo Fisher	Cat# RP88064
LRRTM4-hFc	This paper	N/A

Critical commercial assays		

Heparinase I	Sigma	Cat# H2519
Heparinase II	Sigma	Cat# H6512
Heparinase III	Sigma	Cat# H8891
Chondroitinase ABC	Sigma	Cat# C3667
Protein A agarose	Thermo Fisher	Cat# 20366
Laemmli Sample Buffer	Bio-Rad	Cat# 1610747
4-20% gradient gel	ThermoFisher	Cat# NP0335BOX
Goat anti-Human IgG magnetic beads	Spherotech	Cat# HMS-40-10
Goat anti-human IgG Fc	Jackson Immunoresearch	Cat# 109-605-098

Deposited data		

Mass spectrometry proteomics data	This paper	ProteomeXchange: PXD081065

Experimental models: Cell lines		

Human: HEK293FT	ThermoFisher	Cat# R70007
Rat: embryonic day 18 hippocampal primary neuron culture	This paper	N/A

Experimental models: Organisms/strains		

Mouse: C57BL/6J	The Jackson Laboratory	JAX: 000664
Mouse: *Nrxn1ΔHS*	This paper	N/A
Mouse: *Ptn*^−/−^	RIKEN	B6.129S2-Ptn<tm1Tmu>

Oligonucleotides		

shRNA targeting sequence: Nrx1 Sh: GTGCCTTCCTCTATGACAACT	Zhang et al.^5^	N/A
shRNA targeting sequence: Nrx2 Sh: GAACAAAGACAAAGAGTAT	Zhang et al.^5^	N/A
shRNA targeting sequence: Nrx3 Sh: GGCCAGTGAATGAGCATTA	Zhang et al.^5^	N/A
shRNA targeting sequence: MorB Sh: GGGAAGGGTTGAAGTTTGT	Zhang et al.^5^	N/A

Recombinant DNA		

pLL3.7-V5-Nrxn1α	(Zhang et al.^5^)	N/A
pLL3.7-V5-Nrxn1α-ΔHS	(Zhang et al.^5^)	N/A
pLL3.7-YFP-P2A-V5-Nrxn2α	This paper	N/A
pLL3.7-YFP-P2A-V5-Nrxn2α-ΔHS	This paper	N/A
pLL3.7-V5-Nrxn3α	This paper	N/A
pUCmini-iCAP-PHP-eB	Addgene	103005
pHelper	Agilent	N/A
pAAV-rNrx-TKD	(Cvetkovska et al.^28^)	N/A
pAAV-shMorB	(Cvetkovska et al.^28^)	N/A

Software and algorithms		

Pclamp 10.5	Molecular Devices	https://www.moleculardevices.com/systems/conventional-patch-clamp/pclamp-10-software
Fiji 64-bit (ImageJ2)	National Institute of Health	https://imagej.nih.gov/ij/index.html
GraphPad Prism 10	GraphPad Software Inc	http://www.graphpad.com/scientific-software/prism/
Arivis	Zeiss	https://www.zeiss.com/microscopy/us/products/software/arivis-pro.html

## Highlights

- Pleiotrophin forms a complex with HS-neurexin1 in the brain
- HS and neurexins are required for pleiotrophin’s presynaptic activity
- Pleiotrophin promotes presynaptic assembly and function in the hippocampus
- Loss of pleiotrophin impairs contextual fear discrimination
