# Supplementary material for: Pleiotrophin regulates presynaptic assembly and function through heparan sulfate-dependent binding to neurexin1
Source: Cell Rep. Author manuscript; Available in PMC 2026 Sep 18. (PMC13587102; doi:10.1016/j.celrep.2026.117827)
Supplement: Supplementary file 1 [file NIHMS2206920-supplement-1.pdf]

Cell Reports, Volume *45*

Supplemental information

Pleiotrophin regulates presynaptic assembly and function through heparan sulfate-dependent binding to neurexin1

Qin Xu, Andrew W. Schilling, Alexander W. Sorum, Peipei Zhang, Destini C. Weller, Donovan Whitfield, Gokul Velayoudame, Allison J. Wu, Qian Sun, Linda C. Hsieh-Wilson, and Peng Zhang

Supplemental Figure 1

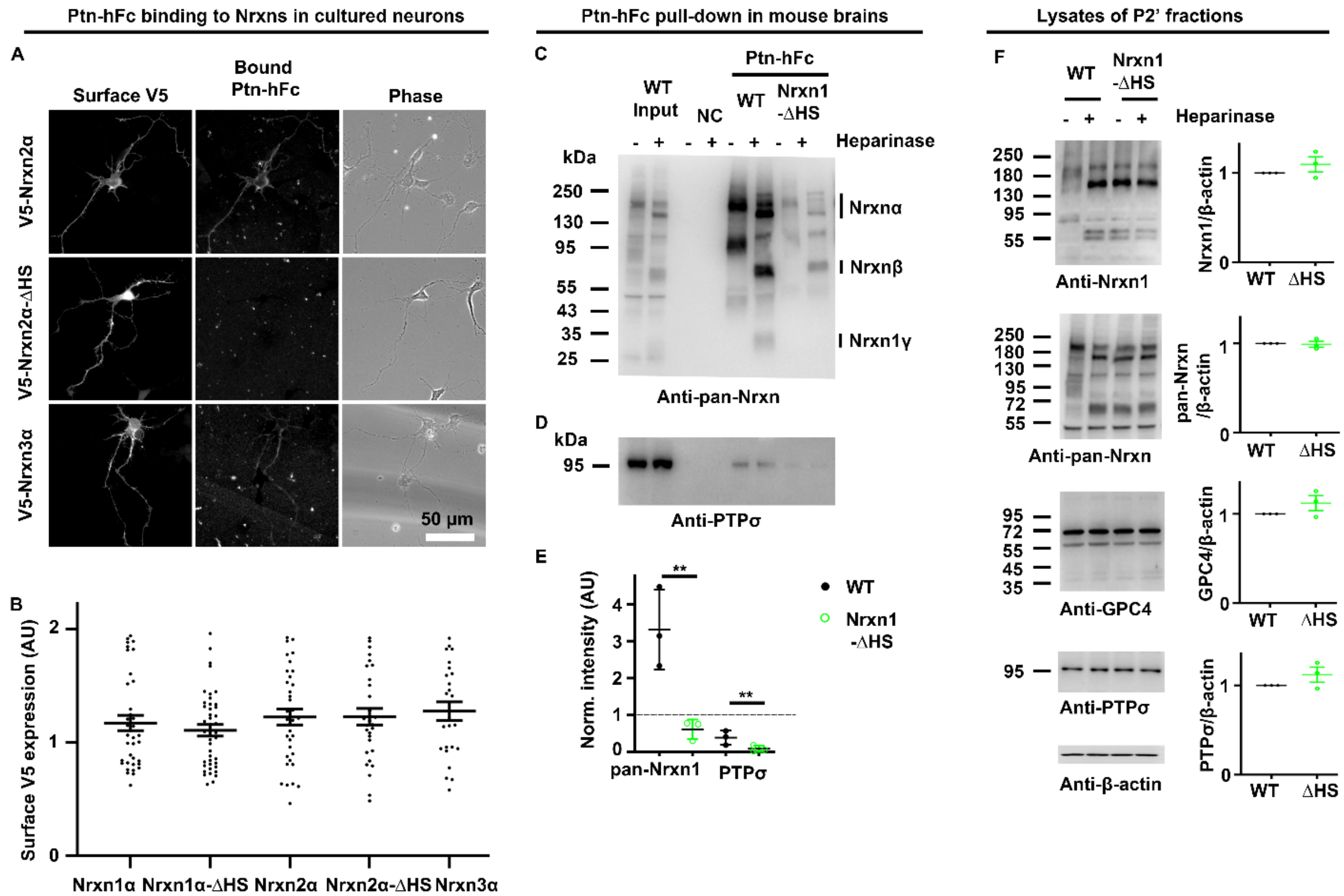

Supplemental Figure 1. Validation of Ptn–hFc binding and pull-down specificity.

(**A**) Representative images of Ptn–hFc binding to V5-tagged Nrxn2α, Nrxn2α-ΔHS, and Nrxn3α expressed in cultured neurons. Surface V5 staining, bound Ptn–hFc, and phase images are shown. Ptn–hFc binding is reduced in the HS-deficient (ΔHS) mutant. Scale bar, 50 µm.

(**B**) Quantification of surface expression of V5-tagged Nrxn1α, Nrxn1α-ΔHS, Nrxn2α, Nrxn2α-ΔHS and Nrxn3α. One-way ANOVA with Bonferroni's test showed no significant differences in surface expression among groups. N = 25-43 cells in 3 independent experiments.

(**C**) Immunoblot analysis of proteins recovered in Ptn–hFc pull-downs from WT and Nrxn1ΔHS mouse brain lysates, probed with anti-pan-Nrxn antibodies. Multiple Nrxn isoforms are detected, and enrichment is reduced in Nrxn1ΔHS samples.

(**D**) Immunoblot of PTPσ in Ptn–hFc pull-downs, showing minimal enrichment compared to Nrxns.

(**E**) Quantification of pan-Nrxn and PTPσ enrichment in Ptn–hFc pull-downs, normalized to input (dashed line). Nrxns show significant enrichment, which is reduced in Nrxn1ΔHS samples. One-way ANOVA with Bonferroni's test, comparing WT input with WT pull-down (pan-Nrxn $p < 0.0001$; PTPσ $p < 0.0001$) and comparing pull-down between WT and ΔHS (**$p < 0.01$); N = 3 independent experiments.

(**F**) Immunoblot analysis of synaptic proteins in P2′ fractions from WT and Nrxn1ΔHS mouse brains, with or without heparinase treatment. Nrxn1, pan-Nrxn, GPC4, and PTPσ levels are comparable between genotypes. Quantification is shown relative to β-actin. N = 3 independent experiments. Error bars, SEM.

Supplemental Figure 2

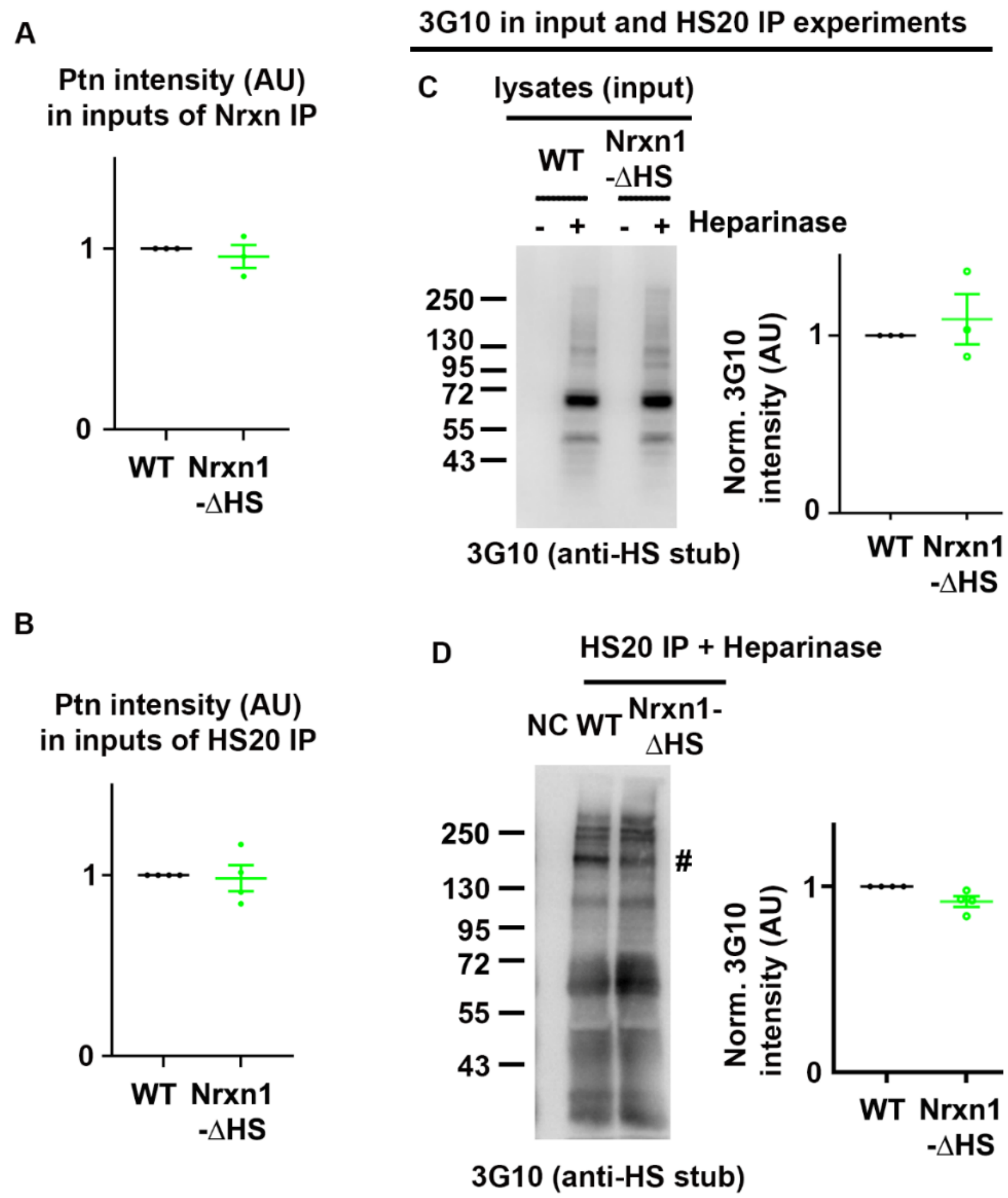

Supplemental Figure 2. Validation of immunoprecipitation and heparinase digestion.

(**A**) Quantification of Ptn levels in inputs of Nrxn immunoprecipitation experiments, showing comparable Ptn expression between WT and Nrxn1ΔHS samples.

(**B**) Quantification of Ptn levels in inputs of HS20 immunoprecipitation experiments, showing comparable Ptn expression between genotypes. Mann-Whitney test; N = 3 independent experiments.

(**C**) Immunoblot of HS stubs (3G10) shows signals in WT and Nrxn1ΔHS brain lysates only after heparinase treatment, confirming efficient digestion of HS chains. Quantification of 3G10 signals is shown. Mann-Whitney test; N = 4 independent experiments.

(**D**) Immunoblot of HS stubs (3G10) following heparinase treatment after HS20 immunoprecipitation. 3G10 signals show comparable HSPGs recovered from HS20 immunoprecipitation between WT and Nrxn1ΔHS samples. Mann-Whitney test; N = 4 independent experiments.

Supplemental Figure 3

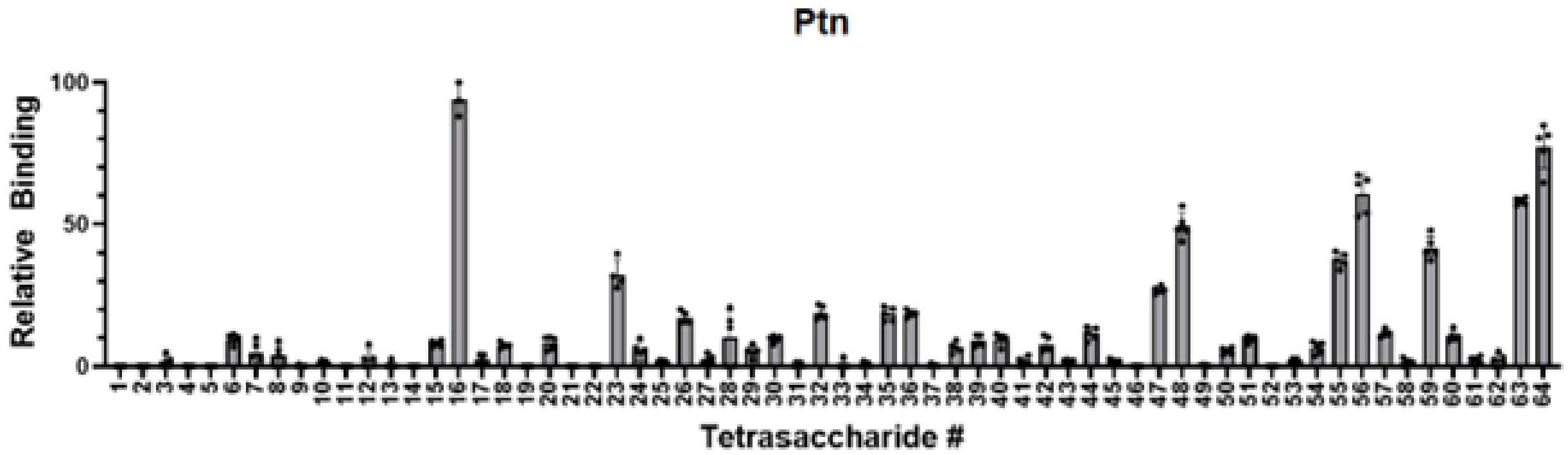

Supplemental Figure 3. Ptn binding to HS oligosaccharides.
Relative binding of Ptn to individual HS tetrasaccharides on a glycan array. Binding intensities are plotted for each structure, highlighting selective interactions with specific sulfation patterns.

Supplemental Figure 4

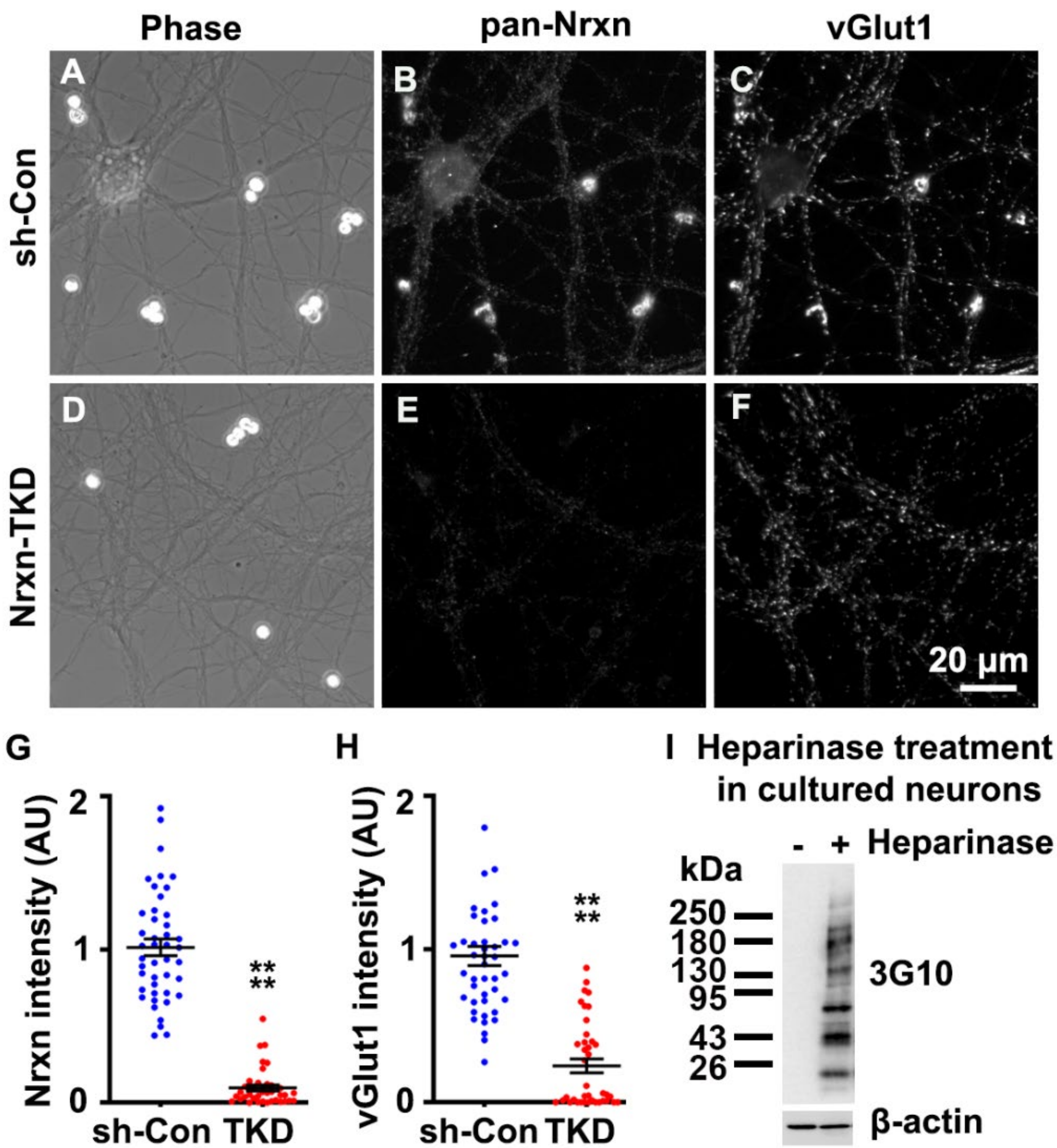

Supplemental Figure 4. Nrxn knockdown impairs Ptn's presynaptic activity and validation of heparinase treatment.

(**A–F**) Representative images of cultured neurons infected with AAVs containing control shRNA (sh-Con) or Nrxn knockdown (Nrxn-TKD), showing phase contrast, pan-Nrxn, and presynaptic marker vGlut1 staining. Nrxn knockdown reduces Nrxn and vGlut1 levels around Ptn-hFc coated beads. Scale bar, 20 µm.

(**G–H**) Quantification of Nrxn and vGlut1 intensities, showing significant reductions in Nrxn-TKD neurons. Mann-Whitney or Welch's test, ****p <0.0001, N= 29-44 from 3 experiments. Error bars, SEM.

(**I**) Immunoblot of HS stubs (3G10) following heparinase treatment in cultured neurons, confirming efficient digestion of HS chains. The 3G10 signal pattern differs from that observed in brain lysates, likely reflecting differences in cellular composition between cultured neurons and brain tissue.

Supplemental Figure 5

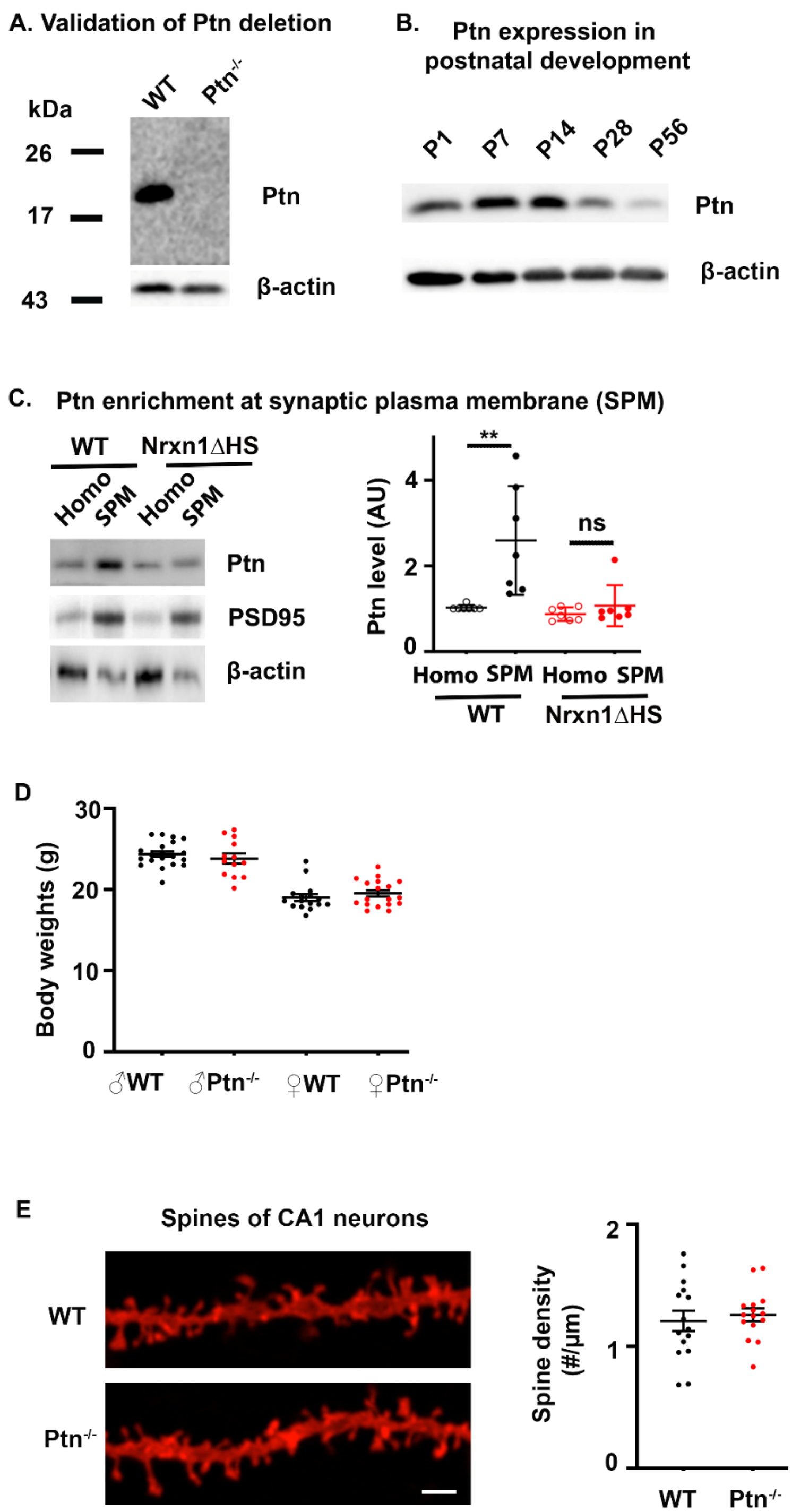

Supplemental Figure 5. Ptn is enriched in synaptic plasma membrane fractions.

(**A**) Immunoblot validation of Ptn deletion in WT and $Ptn^{-/-}$ mouse brains.

(**B**) Developmental expression of Ptn in mouse brain across postnatal stages (P1–P56).

(**C**) Ptn enrichment in synaptic plasma membrane (SPM) fractions is reduced in Nrxn1-ΔHS mouse brains when compared to WT. Ptn signals normalized to WT homogenate (Homo). Two-way ANOVA with Bonferroni's test, $^{**}p < 0.01$; N = 7 mice per genotype.

(**D**) Body weights of Ptn-/- mice were unchanged. Two-way ANOVA with Bonferroni's test: sex effect (P<0.0001), genotype effect (p=0.99). N = 20 male and 11 female WT; 16 male and 18 female $Ptn^{-/-}$ mice.

(**E**) DiI-labeling revealed no alteration in spine density in CA1 pyramidal neurons. Welch's test, P=0.61, N=15 dendrites from 3 WT and 3 $Ptn^{-/-}$ mice. Scale bars, 2 µm. Error bars, SEM.

Supplemental Table 1 HSPGs and CSPGs identified in proteomics

Gene	Relative Spectral Counts	Relative LFQ Intensities
Nrxn1*	1959	944167.6
Nrxn3*	1378	668049.2
Nrxn2*	1270	1525315.388
Gpc1	529	1409381.795
Gpc4	467	591635.534
Tnr	345	645038.524
Agrin	268	126246.1518
Ptprz1	222	381387.7125
Gpc5	212	659392.505
Sdc4	160	147435.1693
Gpc6	159	244414.089
Bcan	152	314968.486
Ncan	133	140562.469
Cspg5	132	436317.152
Vcan	91	31083.9124
Sdc3	86	393063.8683
Gpc3	68	48645.5267

Supplemental Table 2   Top twenty candidates in proteomics

Gene	Relative Spectral Counts	Relative LFQ Intensities
Nrxn1*	1959	944167.6
Nrxn3*	1378	668049.2
Nrxn2*	1270	1525315.388
Ap3b2	734	723912.922
Gpc1	529	1409381.795
Atp1a3	530	610214.39
Gpc4	467	591635.534
Tnr	345	645038.524
Ap3b1	316	178034.7903
Agrin	268	126246.1518
Ptprs	266	208813.256
Ap3d1	247	109898.2846
Ptprd	229	153252.9973
Ptprz1	222	381387.7125
Gpm6a	213	550050.64
Gpc5	212	659392.505
Cask	190	172013.3883
Atp2b4	187	42741.9765
Sdc4	160	147435.1693
Gpc6	159	244414.089
